# Label-free cell based impedance measurements of ZnO nanoparticles—human lung cell interaction: a comparison with MTT, NR, Trypan blue and cloning efficiency assays

**DOI:** 10.1186/s12951-021-01033-w

**Published:** 2021-10-07

**Authors:** Giuseppina Bozzuto, Giuseppe D’Avenio, Maria Condello, Simona Sennato, Ezio Battaglione, Giuseppe Familiari, Agnese Molinari, Mauro Grigioni

**Affiliations:** 1 National Centre for Drug Research and Evaluation, Istituto Superiore di Sanità, Viale Regina Elena 299, 00161 Rome, Italy; 2 National Centre for Innovative Technologies in Public Health, Istituto Superiore di Sanità, Rome, Italy; 3 CNR-ISC Sede Sapienza and Department of Physics, Sapienza University of Rome, Rome, Italy; 4 Department of Anatomical, Histological, Forensic Medicine and Orthopedics Sciences, Sapienza University of Rome, Rome, Italy

**Keywords:** Electric cell-substrate impedance, Nanomaterials, Zinc oxide nanoparticles, Cytotoxicity tests, Confocal microscopy, Electron microscopy

## Abstract

**Background:**

There is a huge body of literature data on ZnOnanoparticles (ZnO NPs) toxicity. However, the reported results are seen to be increasingly discrepant, and deep comprehension of the ZnO NPs behaviour in relation to the different experimental conditions is still lacking. A recent literature overview emphasizes the screening of the ZnO NPs toxicity with more than one assay, checking the experimental reproducibility also versus time, which is a key factor for the robustness of the results. In this paper we compared high-throughput real-time measurements through Electric Cell-substrate Impedance-Sensing (ECIS®) with endpoint measurements of multiple independent assays.

**Results:**

ECIS-measurements were compared with traditional cytotoxicity tests such as MTT, Neutral red, Trypan blue, and cloning efficiency assays. ECIS could follow the cell behavior continuously and noninvasively for days, so that certain long-term characteristics of cell proliferation under treatment with ZnO NPs were accessible. This was particularly important in the case of pro-mitogenic activity exerted by low-dose ZnO NPs, an effect not revealed by endpoint independent assays. This result opens new worrisome questions about the potential mitogenic activity exerted by ZnO NPs, or more generally by NPs, on transformed cells. Of importance, impedance curve trends (morphology) allowed to discriminate between different cell death mechanisms (apoptosis *vs* autophagy) in the absence of specific reagents, as confirmed by cell structural and functional studies by high-resolution microscopy. This could be advantageous in terms of costs and time spent. ZnO NPs-exposed A549 cells showed an unusual pattern of actin and tubulin distribution which might trigger mitotic aberrations leading to genomic instability.

**Conclusions:**

ZnO NPs toxicity can be determined not only by the intrinsic NPs characteristics, but also by the external conditions like the experimental setting, and this could account for discrepant data from different assays. ECIS has the potential to recapitulate the needs required in the evaluation of nanomaterials by contributing to the reliability of cytotoxicity tests. Moreover, it can overcome some false results and discrepancies in the results obtained by endpoint measurements. Finally, we strongly recommend the comparison of cytotoxicity tests (ECIS, MTT, Trypan Blue, Cloning efficiency) with the ultrastructural cell pathology studies.

**Graphic Abstract:**

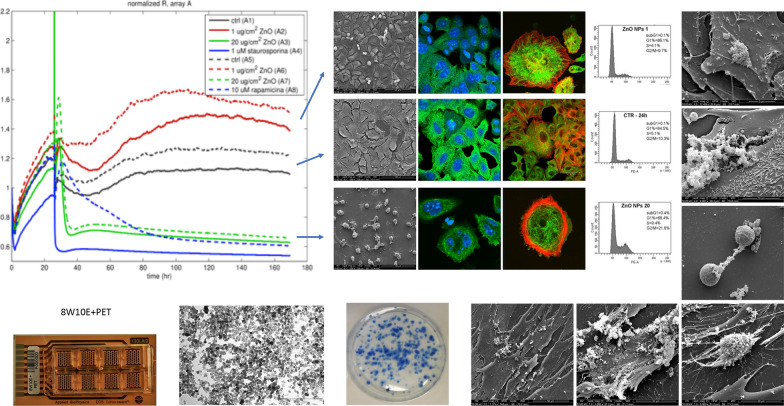

**Supplementary Information:**

The online version contains supplementary material available at 10.1186/s12951-021-01033-w.

## Background

As nanomaterials become part of our daily life, environmental exposure to nanomaterials is inevitable; as a result, nanotoxicity research is gaining increasing attention to verify toxic effects on human health [1]. Because of the larger total surface area to volume ratio, small size, and other physico-chemical properties, nanomaterial can display toxicity profiles that are very different from those of bulk materials of the same composition. Consequently, nanomaterial are generally more toxic than the corresponding fine particles [2].

The importance of developing in vitro tests for toxicity evaluation of nanomaterials is emphasized by the setup of standardized and validated protocols by efforts of international bodies [3, 4]. In agreement with the recommendations of ISO/TR 10993–22:2017 Biological evaluation of medical devices — Part 22: Guidance on nanomaterials: “*Corroboration of several test results from different methodologies might be required for a scientifically sound interpretation*” the main in vitro assays used in nanotoxicology studies are MTT, WST1, LDH assay, trypan blue, and propidium iodide staining [5]. However, traditional cytotoxicity assays have some limits, because nanoparticles (NPs) can interact with the soluble indicators inherent in these assays or interfere with absorbance reading [6]. Moreover, high-precision toxicity tests require a collection of large numbers of data points from multiple experiments (high-throughput test).

Literature data show the widespread application of metallic nanomaterials in the biomedical and pharmaceutical fields [7, 8]. Among these, Zinc oxide (ZnO), a material with semiconducting, piezoelectric, and pyroelectric multiple properties has been used as a nano biomaterial [9–11]. ZnO nanoparticles (ZnO NPs) have many advantages, including the high surface-to-volume ratio, chemical stability, and electrochemical activity. The main biomedical applications of ZnO NPs include antimicrobial activity, anticancer therapy, targeted selective anticancer therapy, drug delivery, tumor detection, and food preservation [12–20].

There is a huge body of literature data on ZnO NPs toxicity. However, the reported results are seen to be increasingly discrepant, and deep comprehension of the ZnO NPsbehaviour in relation to the different experimental conditions is still lacking. Recent literature overview emphasizes the screening of the ZnO NPs toxicity with more than one assay, checking the experimental reproducibility also versus time, which is a key factor for the robustness of the results [21]. Thus, it is recommended, in the assessment of cytotoxicity of ZnO NPs, and more generally of NPs, multiple test methods should be carried out to authenticate the results.

Starting from this need, we performed a comparison between high-throughput test real-time measurements by Electric Cell-substrate Impedance Sensing (ECIS) with endpoint measurements of multiple independent assays (MTT, TB, NR, and cloning efficiency). ECIS is a label-free non-invasive method, based on impedance measurements: it can be used to monitor, in real-time, different kinetic physiological, and morphological characteristics of a culture cell population after exposure to several agents, such as xenobiotics [22, 23]. A noteworthy characteristic of cell impedance measurements is the continuous monitoring of cell viability, so that effects at very different timescales can be revealed. With traditional assays, this is obviously very cumbersome, if not impossible [24–27].

One of the most important ways of entry for ZnO NPs is the respiratory tract. Thus, in our study, a human bronchoalveolar adenocarcinoma cell line (A549) was chosen as a reference model of airway epithelium, because it is frequently used in cytotoxicity testing of NPs [28], and it has been extensively characterized [29]. Gingival fibroblasts (HGF-1) were also used, in order to test ZnO NPs toxicity on a human normal cell model. We evaluated by ECIS measurements the anti-proliferative and cytotoxic effect of low and high doses of ZnO NPs on both A549 and HGF-1 cells, for a short and long time of exposure.

Data obtained by impedance measurements were compared with those obtained by multiple endpoint measurements. In particular we used traditional dye-based tests that analyze different cellular parameters, such as mitochondria functionality (MTT), lysosome homeostasis (Neutral red), membrane permeability (Trypan blue). Cell proliferation capability was also tested by the cloning efficiency assay. We also analyzed the cell death induced by ZnO NPs by Annexin V/PI staining assay and bivariate analysis of DNA cell content versus cyclin B expression. Then, we completed the analysis with ultrastructural studies of the cytoskeleton remodeling by laser scanning confocal microscopy, and of the NPs-cell interaction by high-resolution electron microscopy.

Results obtained in our study demonstrate that high throughput impedance-based cell monitoring can be an efficient alternative cytotoxicity assay to more traditional approaches for testing NPs, allowing to perform a large number of experiments simultaneously with lower sample consumption and in a time-effective manner. Noteworthy, ECIS allowed to better clarify certain long-term effects of low-dose exposure to ZnO NPs, otherwise not detected by the other tests. Cell pathology microscopical imaging allowed us to sustain the functional ECIS data.

## Results

It is recommended, in the assessment of cytotoxicity of ZnO NPs, and more generally of NPs, the use of multiple test methods to authenticate the obtained data. Thus, in our study, we wanted to analytically compare data obtained by high throughput real-time measurements by Electric Cell-substrate Impedance Sensing (ECIS) with those obtained by multiple endpoint measurements: traditional dye-based tests (MTT, neutral red, and trypan blue) that analyze different cellular parameters (mitochondria functionality, lysosome homeostasis, membrane permeability), and cell proliferation assay (cloning efficiency). We also performed ultrastructural cell pathology studies in order to link functional with structural data.

### Nanoparticle characterization

To avoid artefacts due to possible alteration of commercial preparations, ZnO NPs were characterized by Dynamic Light Scattering (DLS), Variable pressure scanning electron microscopy (VP-SEM), and Transmission Electron Microscopy (TEM) before being used for treatments. At VP-SEM observation, ZnO NPs were clearly observed on the polyethersulfone filter surface. They appeared as very small spherical nanoparticles, frequently arranged in clusters, superimposed and easily distinguishable from the filter surface, having a large globular texture (Additional file 1: Figure S1a–d). Measurements by DLS evidenced the presence of nanoparticles with a mean hydrodynamic diameter (Z-Average) of 63 ± 1.6 nm and a polydispersity index of 0.177 (Additional file 1: Figure S1e). The analysis by TEM revealed well dispersed NPs, characterized by a mean diameter of 39.50 ± 11.86 nm (Additional file 1: Figure S1f).

### Electric cell-substrate impedance sensing assay

We performed the label-free analysis by ECIS on both a human bronchoalveolar adenocarcinoma cell line (A549) and human gingival fibroblast cell line (HGF-1) after the treatment with 1, 5, and 20 µg/cm^2^, continuously monitoring cells for up to 5 days. The doses were chosen following literature data. To identify the cell death mechanisms induced by ZnO NPs, we used as reference substances the apoptosis inducer Staurosporine (STS, 1 μM) and the autophagy inducer Rapamycin (Rap, 10 μM). Cells treated with either STS or Rap were seeded in the same array of the ZnO NPs-treated cells.

8W20idf PET arrays were used to analyze a relatively high number of cells. Thus, impedance fluctuations due to micromotion were smoothed out and did not obscure subtle changes in impedance due to the experimental conditions. In our experiments, measurements were performed at the frequency f = 4000 Hz, which was found to yield the maximum change in resistance of the cell-covered electrode [30]. Resistance and capacitance were normalized at respective values for cell-covered electrodes, immediately before treatment.

**Fig. 1:**
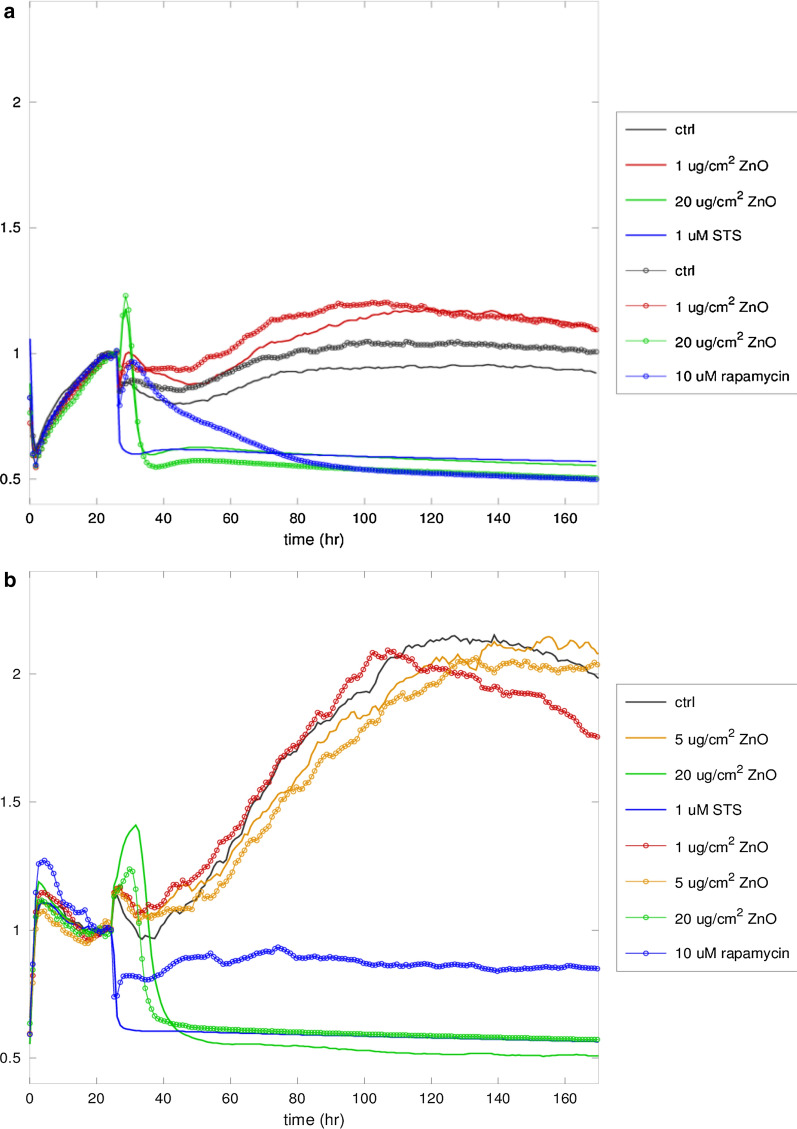
Normalized resistance continuously recorded with ECIS. **a** A549 cell line. **b** HGF-1 cell line. The treatments were delivered 24 h after seeding the wells

Impedance testing (Fig. 1a) showed good reproducibility of results (control and ZnO NPs testing has been carried out in duplicate) since the time-dependent curves (normalized resistance) in the same conditions run nearby along the course of the experiments. The cell layer’s normalized resistance falls off rapidly after treatment at the maximum concentration (20 µg/cm^2^) of ZnO NPs, similarly to the treatment with STS, strongly suggesting an induction of apoptosis by NPs.

On the contrary, resistance and capacity values in cell layers treated with Rap differ remarkably, in the first 60 h after treatment, from the time course of the resistance of STS-treated cells, indicating the different mechanism of cell death i.e. autophagy. Exposure to 1 µg/cm^2^ ZnO NPs elicited a higher resistance increase than controls, suggesting an increase in the metabolic/proliferative activity of A549 cells.

A parallel study with the HGF-1 cell line gave results with high similarity to the ECIS study with A549 cells. First of all, also with HGF-1 cells (Fig. 1b) reproducibile results for duplicate testing (5 and 20 µg/cm^2^ ZnO NPs) were found. Comparing Figs. 1a and b, it can be seen that also with HGF-1 cells the delivery of ZnO NPs at the maximum concentration of 20 µg/cm^2^ elicited a rapid decrease of the normalized resistance (Fig. 1b), even though such decrease was somewhat delayed when compared to A549 cells (Fig. 1a). This suggests that gingival fibroblasts may be more resilient than A549 cells to the presence of ZnO NPs, since the irreversible decay of resistance is observed at a later time, after the treatments.

Another feature in similarity to A549 cell line (Fig. 1a) is the non-inferior resistance increase upon exposure to 1 µg/cm^2^ ZnO NPs when compared to control. This suggests that also for gingival fibroblasts such a concentration of ZnO NPs is not toxic for cells; on the contrary, it seems to elicit a positive metabolic response. At the intermediate concentration (5 µg/cm^2^), slightly lower resistance values than control can be observed, suggesting a limited response (i.e., a slightly impaired cell spreading/attachment on the well) when compared to the NP-free cell layer.

Considering the positive controls, the time course of STS was found to be the same as with A549 cells (an abrupt decrease immediately after treatment). Instead, the delivery of Rap was associated with an immediate decrease of resistance, followed by a sustained recovery (albeit not reaching the pre-treatment values) until the end of the experiments. This result suggests that gingival fibroblasts are also more resilient than A549 cells to autophagic inducers such as Rap. Briefly, curve resistance values obtained by ECIS analysis seem to indicate: (i) a dose-dependent cytotoxic action of ZnO NPs; (ii) a positive metabolic response of the lowest dose of ZnO NPs on both tumour (A549) and normal (HGF-1) cells; (iii) identical curve morphologies of 20 µg/cm^2^ ZnO NPs-treated and STS-treated cells; (iv) different curve morphologies, specific for different cell death mechanism (apoptosis vs autophagy); (v) resilience of HGF-1 normal cells to the ZnO NPs treatment when compared to A549 tumor cells.

### Endpoint measurement multiple assays

To verify points (i) and (ii) reported in ECIS paragraph, we carried out the endpoint measurement multiple assays. In particular, MTT, neutral red (NR), Trypan blue (TB), and colony-forming ability assays were used to evaluate the effects of ZnO NPs on the metabolic response and viability of A549 cells. All the assays were performed after the treatment with ZnO NPs at 1, 5, and 20 µg/cm^2^ for 24, 48, 72, and 144 h. Also in these experiments, A549 cells were treated with the pro-apoptotic agent STS (1 μM), and the autophagic inducer Rap (10 μM).

**Fig. 2:**
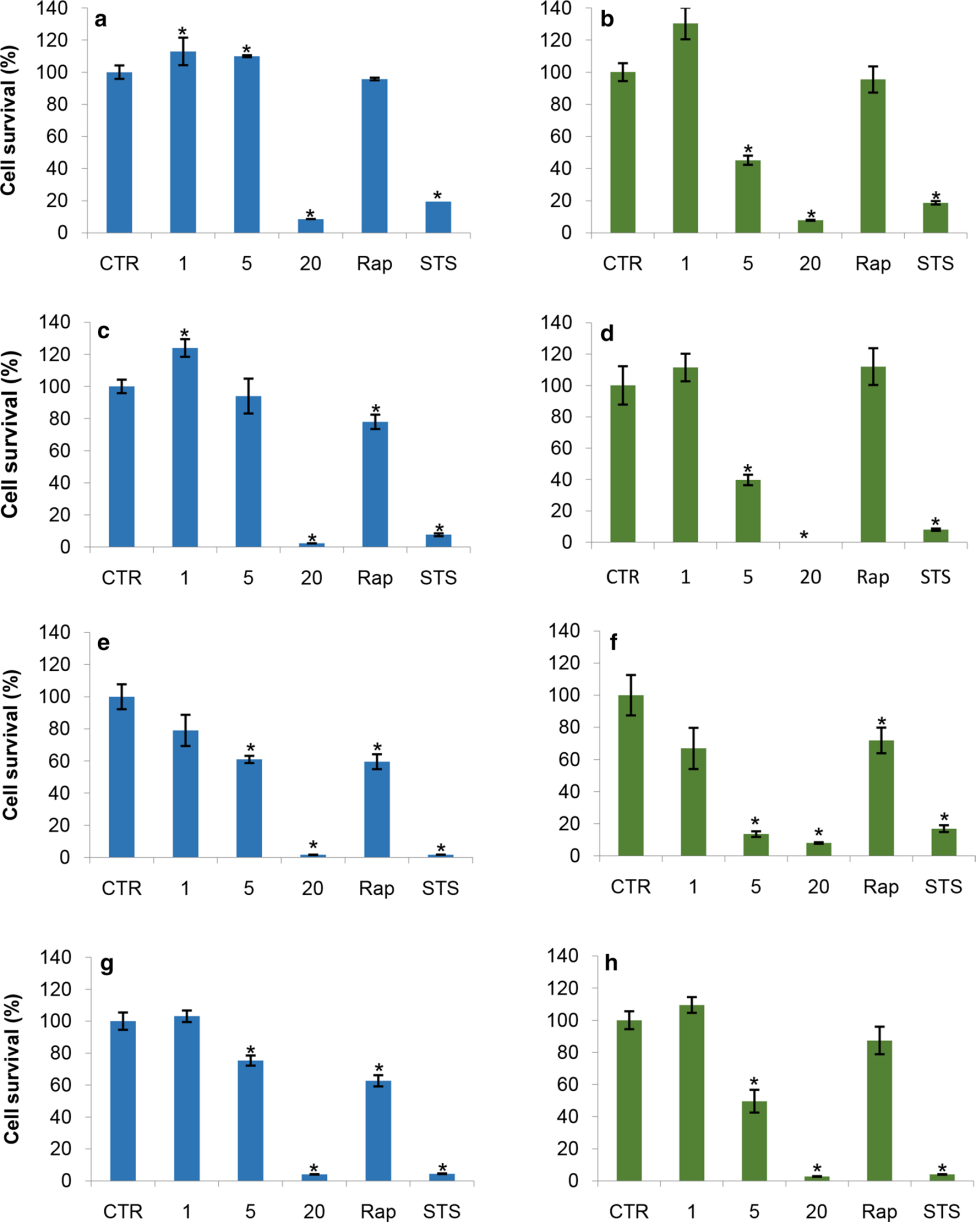
Effects of 1, 5 and 20 µg/cm^2^ ZnO NPs on A549 cell survival. **a**, **c**, **e** and **g** Viability A549 cells evaluated by MTT test after 24, 48, 72, and 144 h of treatment. **b**, **d**, **f** and **h** Viability A549 cells evaluated by neutral red assay after 24, 48, 72 and 144 h of treatment. Rap (10 μM Rapamycin) and STS (1μMStaurosporine) were used as positive control of autophagy and apoptosis induction respectively. Experiments were performed in triplicate; * = p ≤ 0.05; p value Wilcoxon-Mann–Whitney test

Results obtained by MTT (Fig. 2a, c, e, g) and NR assays (Fig. 2b, d, f, h) demonstrated a time- and dose-dependent cell damage induced by exposure to NPs, confirming the results obtained by ECIS assay. MTT assay is based on the 3-[4,5-dimethylthiazol-2-yl]-2,5-diphenyl-tetrazolium bromide conversion by mitochondrial enzymes whereas the NR assay assesses the uptake of neutral red dye by functional lysosomes [31–33].

Strikingly, exposure to 1 µg/cm^2^ ZnO NPs for 24, 48, and 144 h induced an increase of the cellular metabolism (“cell viability” of more than 100% when compared to control), revealed by both MTT and NR tests. These data confirmed the positive metabolic response revealed by ECIS measurements. However, after 72 h of treatment with 1 µg/cm^2^ ZnO NPs a reduction of “cell viability” percentages was revealed by MTT test (− 20%), and by NR test (− 40%). Noteworthy, this reduction was not revealed by the ECIS label-free measurements and this might reflect that the decrease of the absorbance values was very likely due to an engagement of the organelles (i.e. mitochondria and lysosomes) in response to ZnO NPs low doses rather than a loss of cell viability.

After the exposure to 5 µg/cm^2^ ZnO NPs for 24 and 48 h, a noticeable decrease in viable cell percentage was detected by NR test (− 55% and − 60%, respectively) but not by the MTT test (+ 9% and − 6%, respectively). In contrast, cell viability percentages obtained by both assays were comparable after 72 h of treatment with 5 µg/cm^2^ ZnO NPs (− 86% and − 40%, respectively). The discrepancy of these results suggested that the decrease of absorbance recorded with the NR test at 24 and 48 h could be due to an engagement of lysosomes in the initial trafficking of ZnO NPs. Overtime (72 h) the damage induced by ZnO NPs increases, according to both MTT and NR tests and in agreement with ECIS measurements. A partial recovery was recorded at 144 h.

The highest concentration of ZnO NPs (20 μg/cm^2^) induced a dramatic decrease (more than—90%) of cell viability, already after 24 h of treatment, and the cell damage persisted over time. In this case, both MTT and NR data was in agreement with the ECIS measurements trend.

To better clarify the cytotoxic activity of ZnO NPs, we compared the results obtained by NR and MTT tests with those obtained by Trypan blue assay (Additional file 2: Figure S2a–d). This method is based on the principle that living cells have intact membranes, thus they can exclude the dye from their internal space, whereas dead cells do not. In this assay, cells incubated with the dye after the treatment are immediately examined under the microscope to determine the number of living/dead cells. Generally, results obtained by Trypan blue confirmed that, after ZnO NPs exposure, the survival rate of A549 cells underwent substantial decreases in a time- and dose-dependent manner. Data obtained after the exposure to 1 and 5 µg/cm^2^ indicated a low (if any) increase of Trypan blue positive cells. This result seemed to be in agreement with the hypothesis that the decrease of absorbance values detected in some case by NR or MTT test does not always reflect a decrease in cell viability. Conversely, significant uptake of TB was detected by cells treated with 20 μg/cm^2^ very likely due to the membrane “permeabilization” caused by the NP-cell interaction (see below the ultrastructural data on ZnO NP-cell interaction section). Trypan blue assay also confirmed the effects of STS and Rap found with MTT and NR tests.

The proliferative capacity of A549 cells, following treatment with ZnO NPs, STS, and Rap, was evaluated with the clonogenic assay. This assay measures the ability of a single cell to form a colony. It has been reported to be more sensitive than colorimetric assays since in the latter even dying cells can still be metabolically active and thus give misleading results [34]. Furthermore, the clonogenic assay has the advantage of revealing effects on cell proliferation and long-term effects. Cytotoxicity is determined by measuring the relative cloning efficiency of treated cells compared with the cloning efficiency of control cells. In this experimental set-up, the MTT test was executed in parallel.

**Fig. 3:**
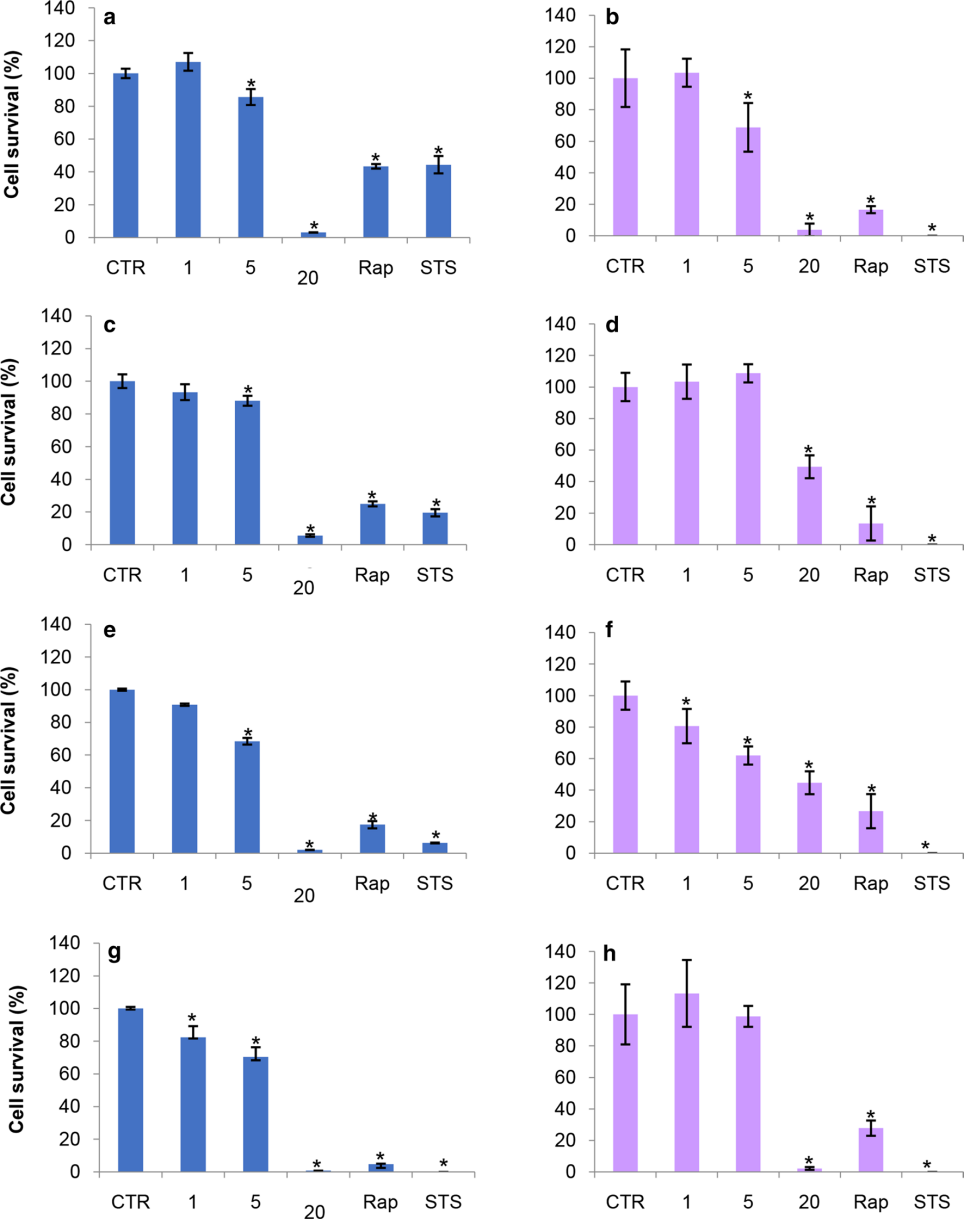
Effects of 1, 5 and 20 µg/cm^2^ ZnO NPs on A549 cell survival. **a**, **c**, **e **and **g** Viability of A549 cells evaluated by MTT test after 24, 48, 72, and 144 h of treatment. **b**, **d**, **f** and **h** Colony numbers of A549 cells analyzed by cloning efficiency assay after 24, 48, 72, and 144 h of treatment. Rap (10 μM Rapamycin) and STS (1μM Staurosporine) were used as positive control of autophagy and apoptosis induction respectively. Experiments were performed in triplicate; * = p ≤ 0.05; p value Wilcoxon-Mann–Whitney test

Data obtained by cloning efficiency (Fig. 3b, d, f, h) showed that the treatment with 1 μg/cm^2^ concentration of ZnO NPs did not interfere with cell survival up to 144 h, indeed it seems to stimulate their proliferation according to ECIS, MTT and NR data. However, according to the results obtained by MTT tests, a reduction (− 20%) in colony-forming ability was detected after 72 h of incubation with 1 μg/cm^2^ ZnO NPs (Fig. 3f).

The treatment with 5 µg/cm^2^ concentration of ZnO NPs induced a significant reduction (about − 30%) in the ability of A549 cells to form colonies after 24 and 72 h (Fig. 3b, f). Similar to the results obtained by MTT and NR experiments, also in this experimental set a restoration of cell proliferation was revealed after 144 h of treatment (Fig. 3h).

The exposure to 20 µg/cm^2^ NPs induced a dramatic reduction of A549 colony number after 24, 48, and 72 h (− 90%, − 45%, and − 50%, respectively) (Fig. 3b, d, f) and the complete inhibition of A549 cell proliferation after the treatment for 144 h (Fig. 3h). In agreement with data obtained by MTT test, Rap and STS inhibited cell proliferation and colony formation capacities already after 24 h of treatment.

The analysis of the endpoint measurements allowed us to confirm (i) the dose-dependent effect of ZnO NPs and (ii) the pro-proliferative effect of the lowest dose of 1 µg/cm^2^ZnO NPs in agreement with ECIS measurements. However, at 72 h endpoint MTT and NR assays revealed a clear alteration in the mitochondrion and lysosome metabolism. Moreover, the clonogenic assay also detected a decrease in the proliferative efficiency of cells treated with the lowest dose. The discrepancy between endpoint measurements and ECIS measurements lead us to reflect on the influence of methodology in the final result. Thus the manipulation of cells necessary to carry out endpoint tests could lead to a misleading interpretation of events. However, the comparison between NR and MTT gave us additional information about the involvement of cell organelles in the cytotoxic action of ZnO NPs.

### Analysis of cell cycle and cell death mechanisms

To verify point (iii) (identical curve morphologies of 20 µg/cm^2^ ZnO NPs-treated and STS-treated cells) reported in ‘ECIS assay’ section, we performed the analysis of the cell cycle and the cell death mechanisms by biochemical assays.

We carried out the analysis of the cell cycle after the treatment with ZnO NPs concentrations ranging from 1 to 20 µg/cm^2^ for 24, 48, and 72 h, by both the univariate analysis of cellular DNA content and the bivariate analysis of cyclin B1 versus DNA content distribution [35].

The first technique is based on the analysis of cellular DNA content after cell staining with propidium iodide (PI), and deconvolution of the cellular DNA content frequency histograms. It reveals the distribution of cells in three major phases of the cycle (G1 vs S vs G2/M) and makes it possible to detect apoptotic cells with fractional DNA content. With this analysis, the effect of ZnO NPs treatment on cell cycle phases was clearly evident after treatment with the highest dose (20 µg/cm^2^) (Additional file 3: Figure S3). The percentage of cells in the G2/M phase noticeably increased in cultures treated for 24, 48, and 72 h. Concerning the other cell cycle phases (subG1, G1, and S) no relevant alterations were revealed by this analysis.

**Fig. 4:**
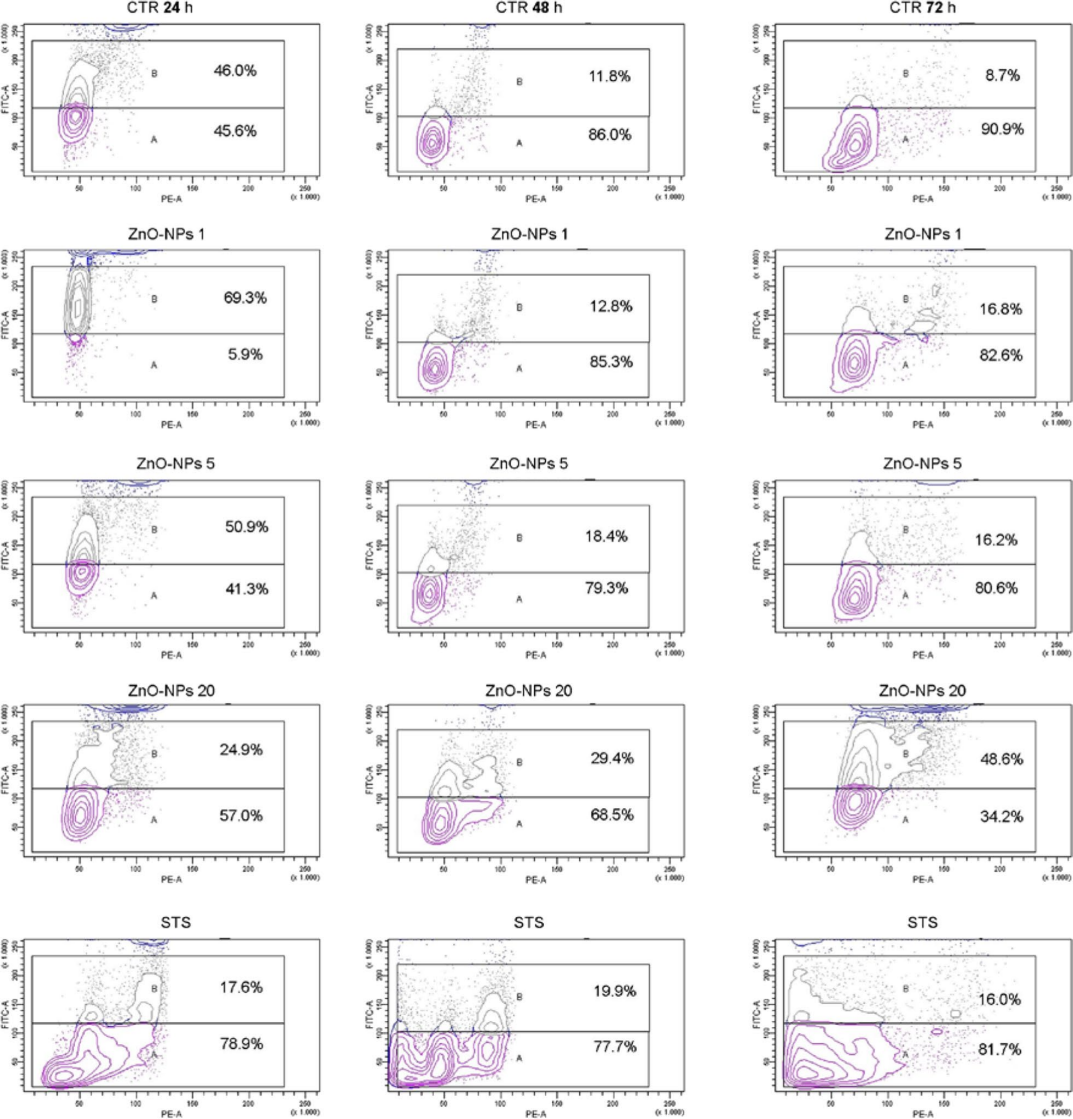
Bivariate analysis of cyclin B1 versus DNA content distribution. A549 cells treated with 1, 5 and 20 ug/cm^2^. STS (1μM Staurosporine) was used as positive control of apoptosis induction

The second technique is based on the bivariate analysis of DNA content and proliferation-associated proteins such as cyclin B1. This approach allows one to distinguish G0 from G1 cells, identify mitotic cells, or relate the expression of other intracellular proteins to the cell cycle position. As shown in Fig. 4, the incubation with low doses of ZnO NPs (1 µg/cm^2^ and 5 µg/cm^2^) induced a slight increase, with respect to control cells, of cyclin B1 expression in cultures treated for 24 h (1.5 and 1.1-fold, respectively), 48 h (1.1 and 1.6-fold, respectively), and 72 h (1.9 and 1.9-fold, respectively). As cells approach mitosis, nuclear cyclin B levels rise which leads to the activation of Cdk1/cyclin B1 complexes. Once activated, Cdk1/cyclin B1 is the major regulator of the transition into and through mitosis [36]. Similarly to STS-treated samples, A549 cells incubated with the highest dose of ZnO NPs for 24 h displayed an initial decrease of the cyclin B1 expression when compared to control cells (0.4 and 0.5-fold, respectively), followed by a relevant increase of the percentage of positive cells at 48 h (1.7 and 2.5-fold, respectively), and at 72 h (1.8 and 5.4-fold, respectively).

Annexin V-FITC assay was carried out to investigate the cell death mechanism induced by the treatment with ZnO NPs (Table 1). The percentages of viable cells decreased with the increase of ZnO-NPs concentrations. Time- and dose-dependent increase of early and late apoptotic cell percentages was revealed. Analysis of apoptotic cell fraction (Table 1) confirmed the highest concentration of ZnO NPs as a strong inducer of programmed cell death already after 24 h of treatment. 1 and 5 µg/cm^2^ ZnO NPs proved to have a quite similar effect on cell viability, except for one-day incubation. These results demonstrated that in these experimental conditions the cell death mechanism induced by ZnO NPs was preferentially apoptosis. This result confirms that the similarity of the morphologies of ECIS curves revealed in samples treated with different substances (i.e. cells treated with STS and cells treated with a high dose of ZnO NPs) is due to the same underlying mechanism, i.e., cell death by apoptosis.

**Table 1:** Results of Annexin V/FITC analysis performed by flow cytometry

	Viable	Early apoptotic	Late apoptotic	Necrotic
24 h				
CTR	86.6 ± 1.5	6.0 ± 0.3	4.1 ± 1.3	3.2 ± 1.0
ZnO 1	87.0 ± 0.3	4.2 ± 0.8	4.1 ± 0.1	4.6 ± 0.3
ZnO 5	74.5 ± 0.9	11.5 ± 1.1	8.2 ± 1.1	5.7 ± 0.9
ZnO 20	51.3 ± 2.8	16.3 ± 1.3	23.0 ± 1.5	9.3 ± 0
Rapamycin	80.0 ± 2.4	3,0 ± 0.8	8.7 ± 0.4	8.2 ± 2.7
STS	72.4 ± 4.4	7.9 ± 0.5	11.9 ± 0.5	7.7 ± 3.4
48 h				
CTR	72.4 ± 1,5	19.9 ± 1.7	4.9 ± 1.5	2.7 ± 1.3
ZnO 1	51.2 ± 5.6	20.3 ± 1.3	17.6 ± 3.0	10.8 ± 7.3
ZnO 5	58.1 ± 5.4	33.5 ± 3.2	9.3 ± 0.3	2.2 ± 1.9
ZnO 20	27.2 ± 2.7	17.2 ± 2.9	55.0 ± 0.4	0.6 ± 0.1
Rapamycin	76.8 ± 2.0	4.2 ± 0.9	16.4 ± 1.6	2.5 ± 1.3
STS	60.4 ± 4.7	9.1 ± 1.0	27.3 ± 4.5	3.1 ± 1.3
72 h				
CTR	78.6 ± 3.8	7.5 ± 1.0	7.0 ± 0.7	6.9 ± 3.5
ZnO 1	46.5 ± 1.3	21.6 ± 1.2	21.5 ± 1.0	10.4 ± 3.5
ZnO 5	51.2 ± 3.2	19.5 ± 2.5	23.7 ± 2.4	5.5 ± 1.6
ZnO 20	25.9 ± 3.5	24.5 ± 3.9	40.4 ± 4.0	9.1 ± 3.4
Rapamycin	73.5 ± 5.2	2.6 ± 0.8	13.1 ± 1.5	11.0 ± 2.5
STS	31.3 ± 7.9	27.7 ± 2.8	35.8 ± 2.8	5.2 ± 2.2
144 h				
CTR	51.3 ± 8.6	12.1 ± 1.8	25.2 ± 4.1	11.4 ± 6.4
ZnO 1	44.3 ± 6.3	10.2 ± 3.0	21.7 ± 2.3	23.6 ± 6.1
ZnO 5	39.9 ± 3.0	12.6 ± 3.6	30.0 ± 1.8	17.4 ± 2.4
ZnO 20	20.0 ± 3.8	2.0 ± 0.9	70.3 ± 6.2	7.6 ± 1.5
Rapamycin	54.0 ± 6.1	10.9 ± 2.0	18.8 ± 3.0	16.2 ± 1.1
STS	51.2 ± 3.1	24.0 ± 3.5	23.4 ± 0.7	1.4 ± 0.3

The values are mean of the cell percentage ± standard deviation of two independent experiments

Thus, the results of cell cycle analysis and cell death mechanisms demonstrated that (i) low doses of ZnO NPs stimulates pro-mitotic levels of cyclin B1; high dose of ZnO NPs induces high level of cyclin B1 and pro-apoptotic signalling; (ii) the cell death mechanism induced by ZnO NPs was preferentially apoptosis; necrosis or autophagy play a secondary role; (iii) unexpected percentages of annexin V positive cells were found in samples treated with 1 µg/cm^2^ and 5 µg/cm^2^ ZnO NPs in disagreement with cytotoxicity test, ECIS assay, and cell cycle analysis. We can hypothesize that the manipulation of cells necessary to carry out the annexin V test, with the associated mechanical stresses, can facilitate the apoptotic events triggered by ZnO NPs, otherwise reversible in still adherent cells. This could lead to a misleading interpretation of events.

### Interaction of ZnO NPs with A549 and HGF-1 cells

**Fig. 5:**
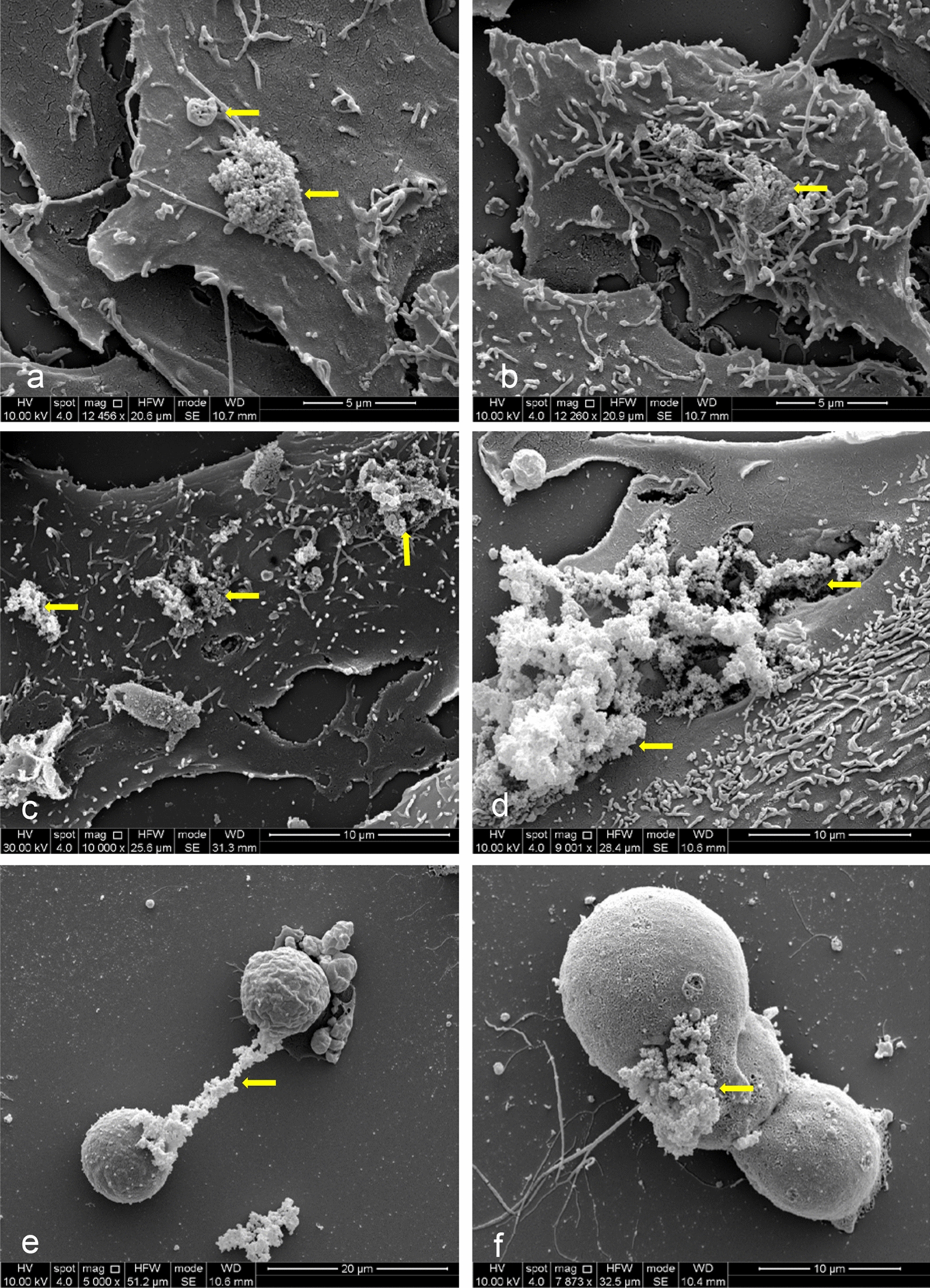
Interaction of ZnO NPs with A549 cells. Scanning electron microscopy observations. **a** and **b** A549 cells treated with 5 μg/cm^2^ ZnO NPs for 24 h. **c** A549 cells treated with 5 μg/cm^2^ ZnO NPs for 48 h. **d** A549 cells treated with 20 μg/cm^2^ ZnO NPs for 24 h. **e** and **f** A549 cells treated with 20 μg/cm^2^ ZnO NPs for 48 h

In order to study how ZnO NPs interact with A549 (Fig. 5a–f) and HGF-1 (Additional file 4: Figure S4a–d) we performed the analysis by high-resolution SEM. The treatments were carried out for 24 h, with 5 and 20 µg/cm^2^ ZnO NPs. In Fig. 5a and b A549 cells treated with 5 µg/cm^2^ZnO NPs for 24 h are shown. During the early stages of interaction cell microvilli get in touch with ZnO-NPs clusters. As the internalization process continues, nanoparticle aggregates, retained by cell microvilli, are engulfed by the cell membrane (Fig. 5c). This engulfment involves making dimples in the cell membrane that deepen with time and eventually seal off. Arrows indicate areas of advanced phase of internalization in which ZnO-NPs clusters are partially sunk into the cell cytoplasm.

In the presence of 20 µg/cm^2^ZnO NPs, large aggregates of NPs interact with the plasma membrane of A549 cells (Fig. 5d) inducing more severe modification of cell morphology. In Figs. 5e and f, actively dividing A549 cells treated with ZnO NPs became blocked and underwent programmed cell death. SEM observations performed on human HGF-1 cells confirmed the interaction with ZnO NPs (Additional file 4: Figure S4a–d). In this case, nanoparticles interacted with both surface and fibrous materials of cells. These observations allowed us to verify (i) that in our experimental conditions the interaction of ZnO NPs with the plasma membrane of target cells did occur; (ii) the state of aggregation of NPs on the plasma membrane; (iii) the active participation of target cells in the process of adhesion and internalization of NPs; (iv) the presence of cells undergoing programmed cell death.

### Remodelling of the cell morphology and cytoskeletal apparatus

The ultrastructural study performed by SEM also evidenced time- and dose-dependent morphological changes in A549 cell cultures treated with 1, 5 and 20 µg/cm^2^ ZnO NPs for 24, 48 and 72 h (Additional file 5: Figure S5). According to data above reported, cultures treated with both 1 and 5 µg/cm^2^ ZnO NPs for 24 h displayed an increase of mitotic cells in comparison with control cultures (arrows). However, after 48 h of treatment with 5 µg/cm^2^ ZnO NPs numerous cells rounded in shape were visible in A549 cultures suggesting a loss of adhesive properties of the cells. The exposure to 20 µg/cm^2^ ZnO NPs induced dramatic modifications of the morphology and cell detachment, suggesting strong alteration of the cytoskeletal structure. This result appeared to be in agreement with the rapid decrease of the normalized resistance measured by ECIS.

**Fig. 6:**
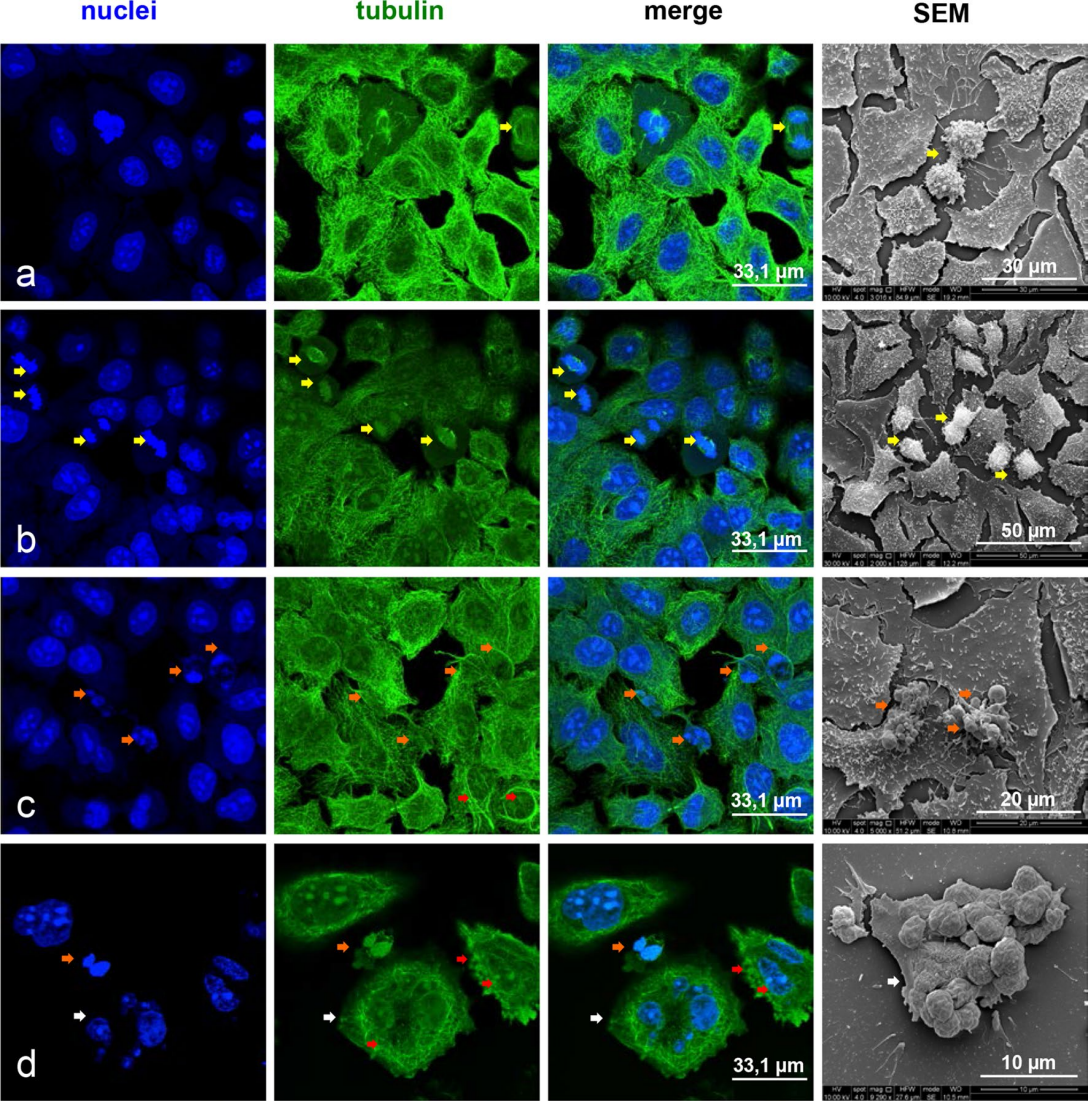
Effects of ZnO NPs treatment for 24 h on microtubule architecture and morphology of A549 cells. Confocal microscopy analysis and scanning electron microscopy observations. **a** Control cells. **b** Cells treated with 1, μg/cm^2^ ZnO NPs. **c** Cells treated with 5 μg/cm^2^ ZnO NPs. **d** Cells treated with 20 μg/cm^2^ZnO NPs

Thus, we performed a confocal microscopy analysis to study the effects of the exposure to ZnO NPs on both microtubule and actin architecture of A549 cells. Cells were treated for 24 and 48 h. The observations confirmed the results obtained by bivariate cell cycle analysis and by SEM ultrastructural study. Indeed, the treatment with 1 µg/cm^2^ ZnO NPs for 24 h induced an increase of the number of dividing cells (Fig. 6b, yellow arrows), when compared to untreated cells (Fig. 6a). No other significant morphological changes were found.

After exposure to 5 µg/cm^2^ZnO NPs for 24 h, we observed the alteration of the microtubule architecture and the presence of apoptotic cells (Fig. 6c, orange arrows). These effects were even more evident in cells treated with the higher concentration of ZnO NPs. In fact, as shown in Fig. 6d, the exposure of cells to 20 µg/cm^2^ ZnO NPs for 24 h caused microtubule collapse (red arrows), chromatin condensation in apoptotic nuclei (orange arrows) and multinucleation (white arrows).

**Fig. 7:**
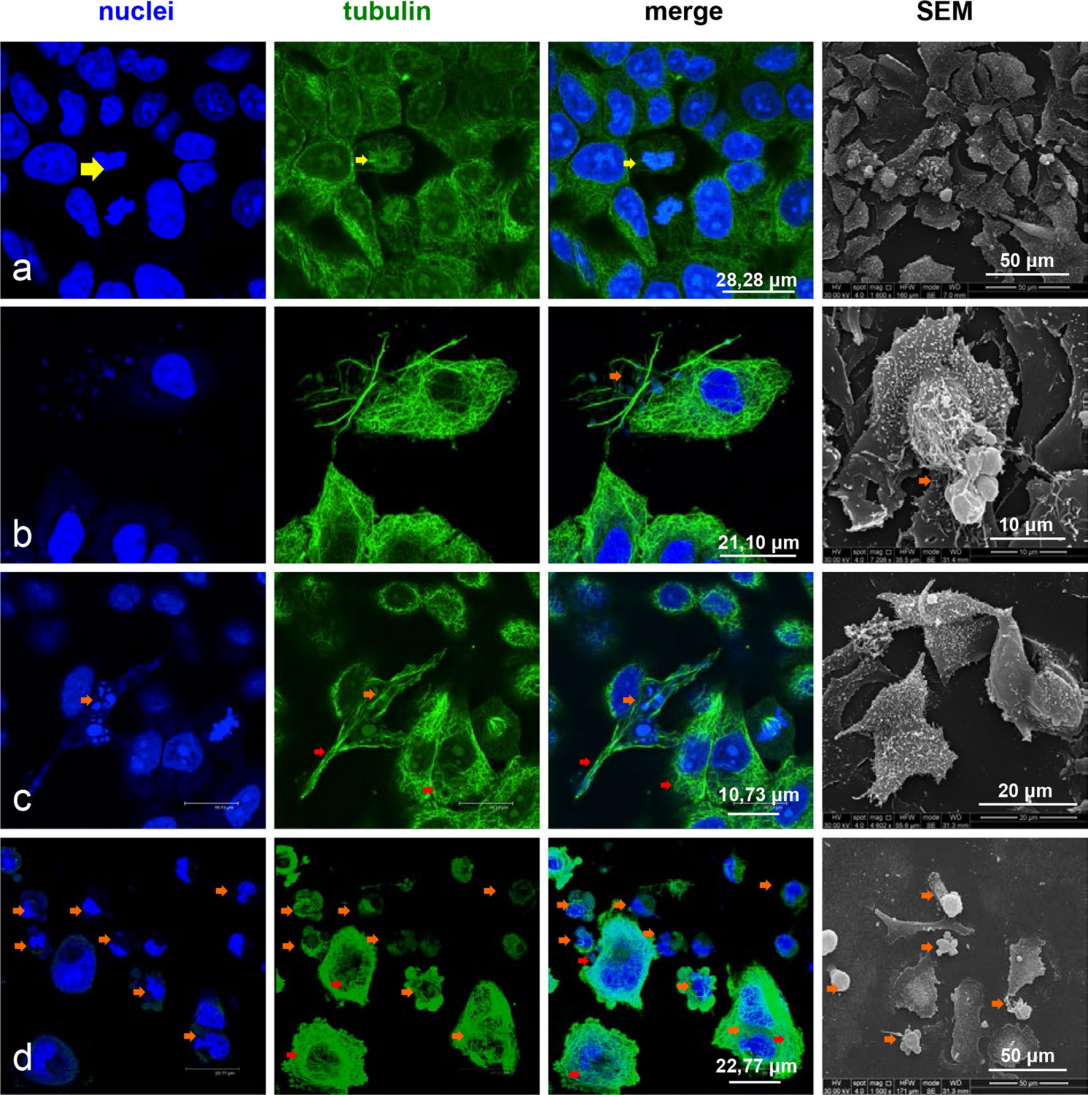
Effects of ZnO NPs treatment for 48 h on the microtubule architecture and on the morphology of A549 cells. Confocal microscopy analysis and scanning electron microscopy observations. **a** Control cells. **b** Cells treated with 1, μg/cm^2^ ZnO NPs. **c** Cells treated with 5 μg/cm^2^ ZnO NPs. **d** Cells treated with 20 μg/cm^2^ ZnO NPs

The incubation with 1 µg/cm^2^ ZnO NPs for 48 h caused an initial alteration of microtubules, detected along with the appearance of few apoptotic cells (Fig. 7b). These effects became more evident in cells treated with 5 µg/cm^2^ and 20 µg/cm^2^ ZnO NP concentrations (Fig. 7c, d). A severe collapse of microtubule architecture and a dramatic increase of apoptotic cells was detected mostly in 20 µg/cm^2^ ZnO NPs treated samples (Fig. 7d).

**Fig. 8:**
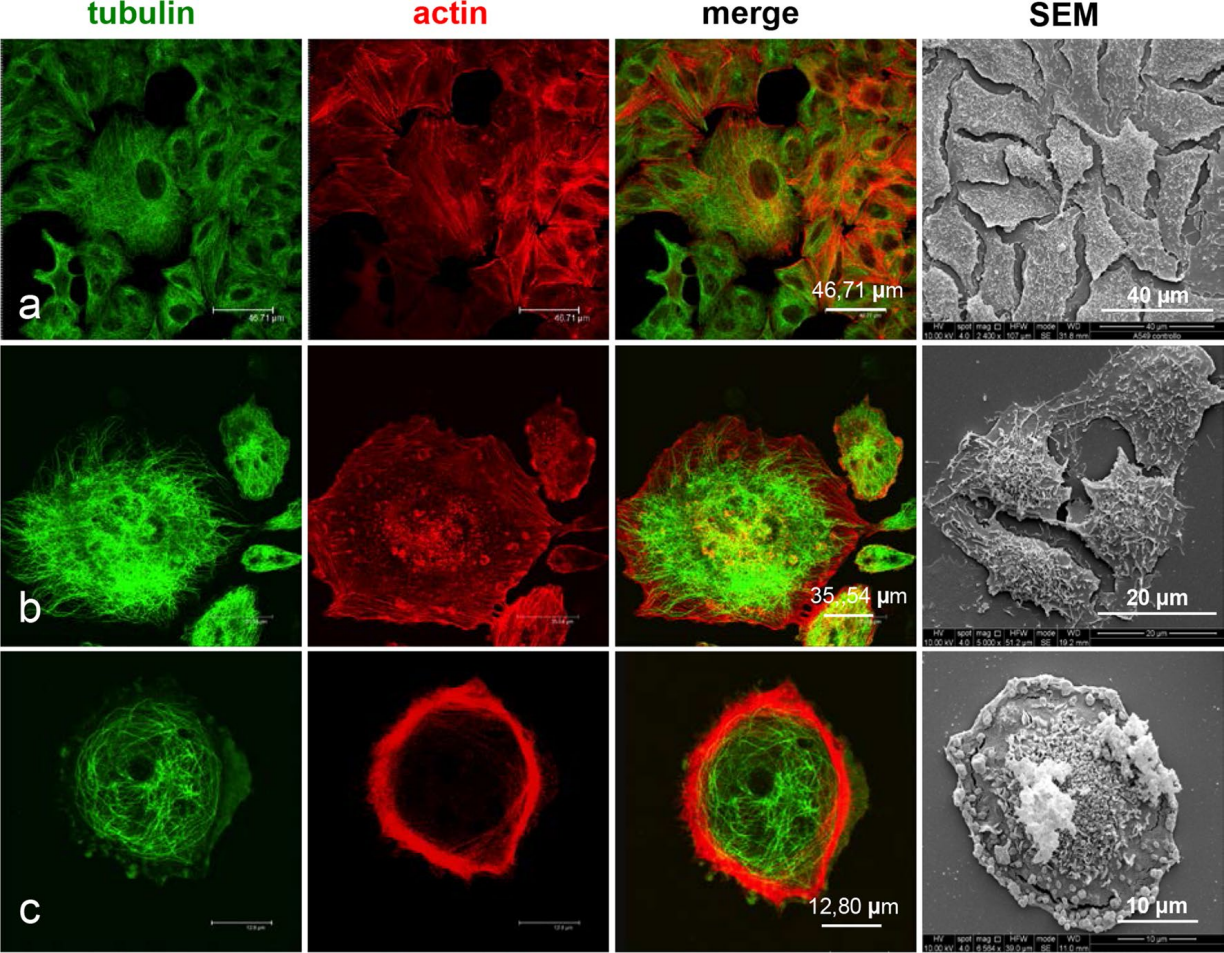
Effects of ZnO NPs treatment for 24 h on actin cytoskeleton and morphology of A549 cells. Confocal microscopy analysis and scanning electron microscopy observations. **a** Control cells. **b** Cells treated with 5 μg/cm^2^ ZnO NPs. **c** Cells treated with 20 μg/cm^2^ ZnO NPs

**Fig. 9:**
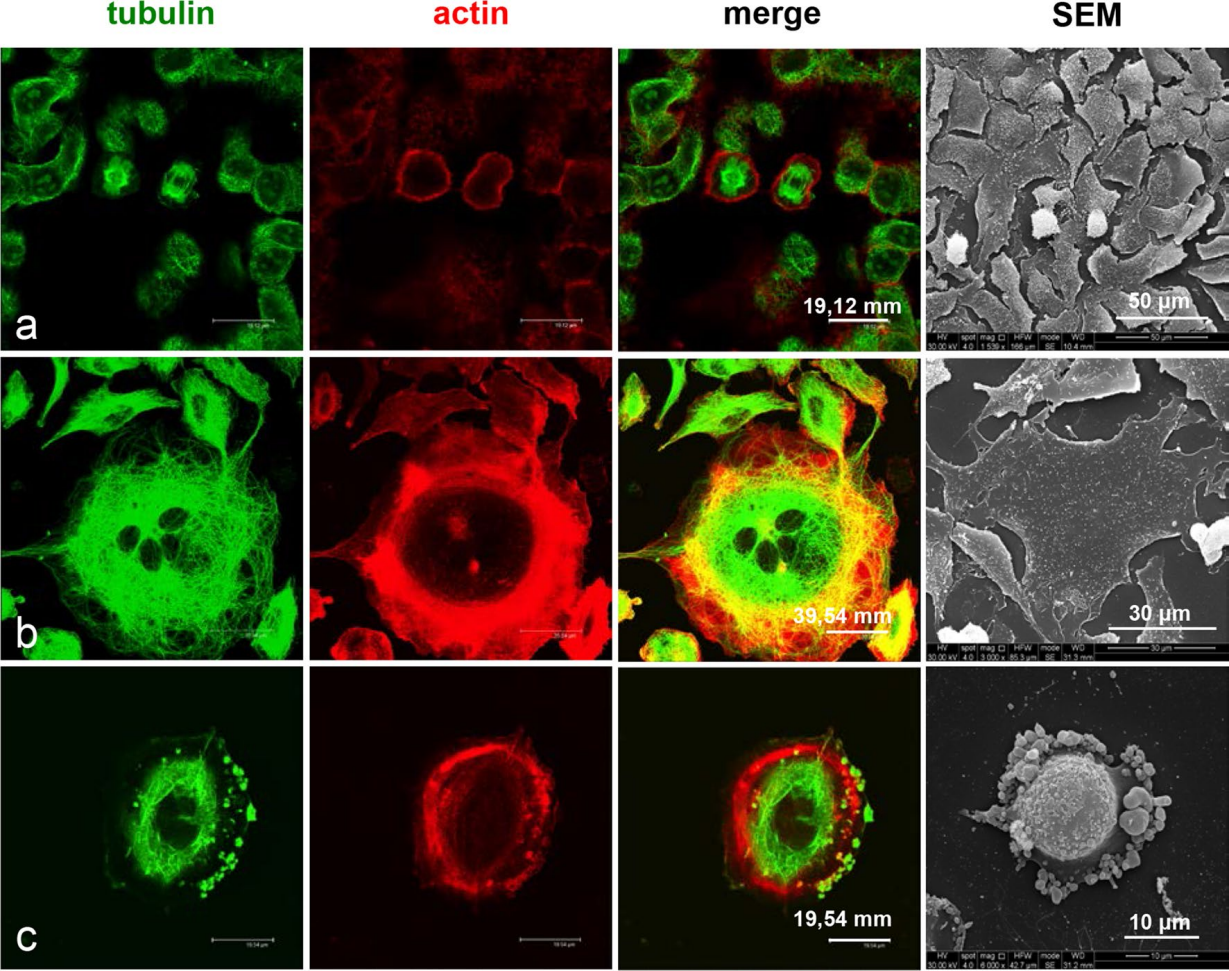
Effects of ZnO NPs treatment for 48 h on actin cytoskeleton and morphology of A549 cells. Confocal microscopy analysis and scanning electron microscopy observations. **a** Control cells. **b** Cells treated with 5 μg/cm^2^ ZnONPs.**c** Cells treated with 20 μg/cm^2^ ZnO NPs

The alteration of the cytoskeletal organization was further confirmed by F actin-specific rhodamine-phalloidin staining. The observations were performed on cultures treated with 5 µg/cm^2^ and 20 µg/cm^2^ ZnO NPs for 24 h (Fig. 8a–c) and 48 h (Fig. 9a–c). As shown in Fig. 9b and c, in addition to the alteration of microtubules (green signal), the treatment with ZnO NPs caused the degradation of F-actin structures (red signal), when compared to untreated cells (Fig. 8a).

In particular, the incubation with 5 µg/cm^2^ ZnO NPs for 24 h (Fig. 8b) induced the initial depolymerization of stress fibers in the perinuclear region. 20 µg/cm^2^ ZnO NPs treatment for 24 h induced agglomeration and marginalization of F-actin (Fig. 8c) which was detected as strongly fluorescent meshwork lining the plasma membrane. Moreover, the microtubule system (green signal) collapsed and strictly intertwined microtubules crowded the region between cell membrane and nucleus. At 48 h after the treatment with20 µg/cm^2^ ZnO NPs, the cytoskeletal disorganization was more evident (Fig. 9a–c). Actin filaments of A549 cells treated with 5 µg/cm^2^ ZnO NPs appeared organized in strong fluorescent rings, whereas microtubules showed to be strongly affected (Fig. 9b). The Increase of ZnO NPs concentration up to 20 µg/cm^2^ induced in several cells intense depolymerization of both actin stress fibres and microtubules, leading to cell retraction and peripheral tubulin-positive blebbing (Fig. 9c).

Actin and tubulin degradation was also confirmed by western blot analysis using anti-β-actin and anti-β-tubulin antibodies. A dose-dependent reduction in the β-actin and the β-tubulin level was observed in lysate obtained from ZnO-NP-treated A549 cells, whereas the level of GADPH remained unchanged indicating that ZnO NPs can specifically degrade cytoskeletal proteins (data not shown).

Thus, the results obtained by the analysis of the cytoskeletal elements indicate: (i) the induction of mitosis by low doses of ZnO NPs, in agreement with the pro-proliferative activity of ZnO NPs above discussed; (ii) the presence of apoptotic cells even in cultures treated with 5 µg/cm^2^; (iii) the damage to microtubules and actin induced by ZnO NPs already visible at sub-cytotoxic doses; (iv) the alteration of cell division with the induction of multinucleated cells.

## Discussion

Metal oxides are the largest class of commercially produced nanomaterials, with a widespread field of applications [37] including memory devices, bulk fillers, molecular nanoelectronics and nanomotors, robotic navigation, low threshold lasers, solar cells, photovoltaic cells, cosmetic formulations, imaging agents and drug delivery systems [38]. In this panorama, human exposure becomes inevitable and risks to human health also increase owing to the high penetrating potential and high reactivity of nanoparticles [39]. Thus, there is an urgent increasing need for new standardized procedures for testing metal oxide nanoparticles toxicity. The importance of developing new in vitro tests is emphasized by the common efforts of international bodies to setup standardized and validated protocols for toxicity evaluation of nanomaterials [3, 4] taking into account that traditional cytotoxicity assays have some limits, because nanoparticles can interfere with absorbance reading thus altering the final results [6], medical devices incorporating or consisting of nanomaterials fall under the highest risk class, class III (when there is a high or medium potential for internal exposure), according to the Regulation (EU) 745/2017, and should be subject to severe controls. A critical revision of the existing methodologies for testing NPs will help us to find stricter evaluation procedures.

Among metal oxide nanoparticles, ZnO NPs are popularly employed in a great variety of biochemical fields and especially as antibacterial agents, anticancer drug/gene delivery, cell imaging, biosensing, and so on [40, 41]. ZnO NPs show cytotoxicity for both prokaryotic and eukaryotic cells and many studies demonstrated their selective toxicity also towards cancer cells. However, with the increasing number of publications, it is observed an increase in the discrepancies between the various reported results. Thus, the screening of ZnO NP toxicity with multiple test methods is recommended [21].

High-throughput screening (HTS) originating from drug discovery provides an opportunity for large-scale testing of nanomaterials. Several different types of HTS methods have been developed and adapted for nanomaterials during the last few years, including diverse methods for label-free cellular screening of nanomaterial uptake, cytotoxicity, and genotoxicity, as well as high-content immunochemical analysis and high-throughput flow cytometry applicable to various biomarkers for, e.g., immunotoxicity, oxidative stress or fibrogenesis [42–46]. Literature data report on real-time monitoring of cellular nanoparticle uptake using electric cell-substrate impedance (ECIS) sensors that measure the alternating current (ac) impedance between the small sensing electrode and the large counter electrode while the cells are cultured on the gold sensing electrode. The cells attach and spread out on the surface of the sensing electrode, thus hindering current flow and causing the electrode impedance to be affected by the shape, adhesion, or mobility of the adherent cells [47–49].

In our study, we used ECIS measurements for the online monitoring of ZnO NPs effects on human lung adenocarcinoma epithelial A549 cells. For their metabolic and macromolecular processing A549 cell line represents an established in vitro robust model to study alveolar type II cellular response to metal oxides injury [29]. As normal counterpart, the human fibroblast HGF1 cell line was employed in ECIS experiments.

Besides, as elsewhere recommended [21] and in agreement with the recommendations of ISO/TR 10993–22:2017, we also evaluated the cytotoxicity of ZnO NPs by endpoint measurements in multiple independent assays. Specifically, results obtained by ECIS measurements were critically compared with those obtained by traditional cytotoxicity tests such as MTT, NR, Trypan blue, and cloning efficiency assays, in order to verify if discrepancies between data obtained by multiple tests did exist. Moreover, the analysis of cell death mechanisms induced by ZnO NPs and ultrastructural pathology studies were performed.

Curve resistance values obtained by ECIS analysis showed a dose-dependent cytotoxic action of ZnO NPs. After the addition of ZnO NPs to A549 and HGF1 cells, the impedance signal increased during the first 10 h before it decreased again at 40 h. Then the signal rose again in both control and surprisingly in 1 µg/cm^2^ ZnO NPs treated samples. The analysis of impedance values of both tumour (A549) and normal (HGF-1) cell lines suggested a cell proliferation triggered by the lowest dose of ZnO NPs (1 µg/cm^2^). Instead, the trend of impedance values at 5 µg/cm^2^ showed a slowdown of the cell growth of A549 tumour cells, but interestingly not of that of HGF-1 normal cells.

After the treatment with either the highest dose of ZnO NPs, or with STS, or Rap, the impedance values dramatically dropped down in both A549 and HGF1 cells. Noteworthy, impedance value curves of cultures treated with 20 µg/cm^2^ ZnO NPs showed the same trend as those revealed in samples treated with STS (apoptosis inducer). Of note this was true both for A549 cells and HGF1 cells. Moreover, ECIS measurements generated distinguishable outputs specific of the different cell death mechanisms (apoptosis *vs* autophagy). Indeed, A549 and HGF1 cultures treated with Rap (autophagy inducer) displayed impedance curves completely different in morphology from those of apoptotic dying cells.

HGF-1 normal cells appear to be more resilient to the ZnO NPs treatment when compared to A549 tumor cells, in accordance with literature data showing that the ZnO-NPs treatment’s susceptibility directly correlate with cell proliferation [50].

The analysis of the endpoint measurements allowed us to confirm both the dose-dependent effect of ZnO NPs and the pro-proliferative effect of the lowest dose of 1 µg/cm^2^ ZnO NPs in agreement with ECIS measurements. The induction of a mitogenic response was confirmed by the increase of mitotic cell number observed in the same samples by both immunofluorescence microscopy and by the analysis of cell cycle. In particular, results obtained by bivariate analysis of cyclin B1 versus DNA content distribution demonstrated that low doses of ZnO NPs stimulate pro-mitotic levels of cyclin B1 [36]. This result appears to agree with model predictions for the mechanism of metal-oxide-nanoparticle-induced stress in A549 cells [51]. It was postulated that mitogenic response induced by ROS generated by a low-exposure dose of metal-oxide nanoparticles and involving several signalling cascades such as MAP kinase pathway elicits several processes such as cellular growth, proliferation, differentiation, transformation and repair mechanisms [51, 52]. On the other hand, high dose of ZnO NPs seem to sustain the inappropriate activation of Cdk1/cyclin B1 that play the opposite role and mediates pro-apoptotic signalling in response to mitotic arrest [53].

However, some discrepancies were found in endpoint measurements (MTT, TB, NR) and cloning efficiency when compared to ECIS measurements. Indeed, ECIS could follow the cell behaviour continuously and noninvasively for hours, so that certain long-term characteristics of cell attachment and spreading (or transient detachment) were accessible, that were not considered in the traditional assays. The label-free technique was particularly useful in our study since, as above reported, nanoparticles can interact with soluble indicators used in traditional assays (as per EN ISO 10993–5). Both false-positive [54] and false-negative results [55] were found in the cytotoxicity assays performed with MTT, in presence of nanoparticles.

From cytotoxicity assays (endpoint measurements) carried out in our study, it was found that the lowest concentration (1 µg/cm^2^) of ZnO NPs did not interfere with cell survival and metabolism except for 72 h time point, at which MTT, NR, and cloning efficiency revealed a decrease of metabolic activity and proliferative capability. At the same endpoint, annexin V test revealed the increase of apoptotic cell percentage (Table 1). The discrepancy between endpoint measurements and ECIS measurements lead us to reflect on the influence of methodology in the final result. In particular, ECIS impedance value curves, which showed a pro-proliferative effect of ZnO NPs at 1 µg/cm^2^, could be explained by the fact that A549 and HGF1 cells analyzed by impedance testing (and not detached as for endpoint measurements of MTT, NR, cloning efficiency) were able to repair the damage induced by ZnO-NPs low-dose exposure and activate Cdk1/cyclin B1 complexes. In the case of normal cells this is extremely important in the assessment of the real toxicity of nanomaterials, on the other hand, it opens worrisome questions about the pro-mitogenic activity of low doses of metal oxide nanoparticles towards transformed tumor cells. Checking the experimental reproducibility also versus time we observed that at 144 h both ECIS measurements and cloning efficiency data demonstrated the pro-mitogenic activity of 1 µg/cm^2^ ZnO NPs. And that is of note.

According to ECIS measurements, the cytotoxicity as detected by either MTT or NR, was induced within 48 h by the treatment with 5 µg/cm^2^ ZnO NPs and the intensity was more pronounced after 72 h and 144 h exposure. However, after treatment with 5 µg/cm^2^ concentrations, different results in MTT and NR assays were shown. In particular, NR test revealed a greater effect compared with that revealed by the MTT test. These results are not contradictory, considering the principles of the assays. MTT assay is based on MTT conversion by mitochondrial enzymes whereas the NR assay assesses the neutral red dye uptake by functional lysosomes [31–33, 56]. The obtained data are in agreement with our previous work where it was demonstrated that ZnO NPs, after entering cells, localize in the cytoplasm, mitochondria, and in structures such as lysosomes [57].

A steep decline in cell viability was seen at 20 µg/cm^2^ of ZnO NPs treatment at all times of exposure bothwith ECIS, and endpoints measurements (MTT, NR, and cloning efficiency tests). As well documented in literature data higher toxicity of ZnO NPs is attributed to its high solubility in the extracellular region which will, in turn, increase intracellular [Zn2 +] level or it may be due to the direct entry of NPs into the cell, causing an increase of intracellular [Zn2 +] level [58].This will alter the activity of Zn-dependent enzymes and transcription factors. This event could be the crucial factor responsible for the ZnO NPs induced cytotoxicity. Moreover, zinc ions induce the damage mitochondria and lysosomes, further disrupting the negative feedback mechanisms between ROS and mitophagy, leading to damaged mitochondria accumulation, and excessive ROS production [59].

As far ultrastructural cell pathology studies are concerned, one of the first responses to external stimuli such as nanoparticles includes changes in cell morphology following NPs-surface interaction, internalization, and enzymatic and signaling pathways. High-resolution SEM images of A549 and HGF1 monolayers clearly indicate the co-operation of cell structures (microvilli) in the active internalization of ZnO NPs. According to cytotoxicity and ECIS measurements, no evident morphological changes were seen in A549 and HGF1 cell lines treated with the lowest dose of ZnO NPs. On the other hand, both SEM and confocal microscopy revealed the increase of the number of mitotic cells. These observations allow us to exclude the cell flattening as the cause of the increase of impedance values.

On the contrary, exposure to 5 µg/cm^2^ ZnO NPs induced depolymerization of F-actin in the perinuclear region and consequent marginalization and flattening of A549 cells. Moreover, the collapse of tubulin architecture was seen.

The evident catastrophic change of morphology and cytoskeletal apparatus was observed after exposure to 20 µg/cm^2^ ZnO NPs after 24 h treatment. In particular we found microtubule collapse, chromatin condensation in apoptotic nuclei, and multinucleation. Cells retracted into a round shape and got detached from the surface of the culture flask, in agreement with the steep dropping of impedance values revealed by ECIS and with the block in G2/M phase found by the univariate analysis of cellular DNA content.

Actin is an essential cellular cytoskeleton component which is a zinc scavenging protein involved in migration, cell division, and cellular architecture maintenance. Overexpression of Rac1 and Rho A at the mRNA level was reported after ZnO-NPs exposure [60]. Small GTPases such as RhoA, Rac1, and Cdc42 are critical for triggering actin reorganization thus modulating cellular morphology. Several reports indicate that cytoskeletal collapse exerted by ZnO NPs causes triggering of ROS-dependent apoptosis [61–63].

## Conclusions

Recent literature overview emphasizes the screening of the ZnO NPs (or more generally NP) toxicity with more than one assay [21]. In agreement with the recommendations of ISO/TR 10993–22:2017 Biological evaluation of medical devices—Part 22: Guidance on nanomaterials: “Corroboration of several test results from different methodologies might be required for a scientifically sound interpretation”, the main aim of this study was to compare ECIS measurements with endpoint measurements in multiple independent assays to analyze cytotoxicity of NPs. In our tests ZnO NPs were chosen as NPs model because there are widely employed in biomedical fields and in order to accomplish recent literature challenges [21]. Specifically, results obtained by ECIS measurements were compared with those obtained by traditional cytotoxicity tests such as MTT, NR, Trypan blue, and cloning efficiency assays. Moreover, analysis of cell death mechanisms induced by ZnO NPs and ultrastructural cell pathology studies were performed.

We found discrepancies between data from endpoint measurements and those from ECIS measurements. This lead us to reflect on the importance and influence of the methodology employed in the final result. We found that the manipulation of cells necessary to carry out endpoint tests could lead to a misleading interpretation of events. Differently from the endpoint measurements by MTT, TB, NR, and cloning efficiency used to study adherent A549 cell cultures under treatment with ZnO NPs, ECIS could follow the cell behaviour continuously and noninvasively for hours, so that certain long-term characteristics of cell proliferation were accessible. Moreover, false-positive and false-negative results revealed in dye-based cytotoxicity assays could be avoided. On the other hand, comparison between NR and MTT gave us additional information about the involvement of cell organelles in the cytotoxic action of ZnO NPs.

ECIS measurements were particularly important for the revealing of the pro-mitogenic activity of low-dose ZnO NPs exposure. A pro-mitogenic effect is extremely important for the assessment of the real toxicity of nanomaterials in the case of normal cells. It also opens new worrisome questions about the pro-mitogenic activity potentially exerted by NPs on dormant transformed cells.

Impedance curve trends (morphology) gave specific information about the cell death mechanism by discriminating between apoptosis and autophagy. This could be advantageous in terms of sensitivity, costs and time spent. Indeed, ECIS can avoid false apoptotic deaths induced by the detachment of cells necessary for endpoint measurements, reduces the cost for specific reagents and limits the time spent for additional experiments (i.e. apoptosis/autophagy specific tests).

Ultrastructural studies completed our study and allow to elucidate the mechanism of action of ZnO NPs on actin and tubulin organization of A549 cells. Actin filaments are one of the critical elements of the cell division machinery ZnO NPs at highest dose (20 µg/cm^2^) induced severe microtubules collapse, undermining the ability of A549 cells to carry out essential functions like movement and division. Actually, a block in G2/M phase was clearly detected. Any tampering with microtubule dynamics during cell division produces abnormal spindles leading to apoptosis or unequal chromosome distribution in the daughter cells. ZnO NPs-exposed A549 cells showed an unusual pattern of actin and tubulin distribution which might trigger mitotic aberrations leading to genomic instability [60, 62].

At the end of our study we can conclude that ZnO NPs toxicity can be determined not only by the intrinsic NPs characteristics, but also by the external conditions like the experimental setting.

In Table 2 we report the contribution of the single experimental assay in the understanding of cytotoxic mechanism of ZnO NPs. ECIS test based on the measurement of electrical impedance in real-time has the potential to recapitulate the needs required in the evaluation of nanomaterials by contributing to the reliability of cytotoxicity tests. Moreover, it can overcome some false results and discrepancies in the results obtained by endpoint measurements. Finally, we strongly recommend the comparison of cytotoxicity tests (ECIS, MTT, Trypan Blue, Cloning efficiency) with the ultrastructural cell pathology studies.

**Table 2:** Contribution of single experimental assays

	ECIS	MTT/NR/TB/CE assays	Cell cycle/cell death assays	Immunofluorescence	SEM
Pro-mitogenicactivity	+	+		+	+
Cytotoxicity	+	+	+	+	+
Cell death	+		+	+	+
Alteration of Cytoskeleton	+			+	+
Alteration of morphology	+			+	+

## Methods

### Aim, design and setting of the study

It is recommended, in the assessment of cytotoxicity of ZnO NPs, and more generally of NPs, the use of multiple test methods to authenticate the obtained data. Thus, the main aim of this study was to analytically compare data obtained by high throughput real-time measurements by Electric Cell-substrate Impedance Sensing (ECIS) with those obtained by multiple endpoint measurements: traditional dye-based tests (MTT, neutral red, and trypan blue) that analyze different cellular parameters (mitochondria functionality, lysosome homeostasis, membrane permeability), and cell proliferation assay (cloning efficiency). We also performed ultrastructural cell pathology by laser scanning confocal microscopy and high resolution scanning electron microscopy in order to link functional with structural data. In order to compare data from ECIS and endpoint measurements specific experimental setting were carried out (see Additional file 6).

### Materials

DMEM medium, RPMI 1640 medium, penicillin/streptomycin solution, l-glutamine solution 200 nM and non essential amino acids 100X were purchased from EuroClone S.p.A. Fetal bovine serum (FBS) was purchased from Hyclone. Anti-α and β tubulin antibodies, DMSO (Dimethyl sulfoxide), MTT (3-[4,5-dimethylthiazol-2-yl]-2,5-diphenyl-tetrazolium bromide solution), phalloidin TRITC, Phosphate-buffered solution (PBS), Rapamycin, ribonuclease (RNAse), Staurosporine, Triton X-100 and ZnO NPs were purchased from Sigma Aldrich. Annexin V-FITC apoptosis detection kit was purchased from BD Bioscience, Becton, Dickinson & Company. Propidium iodide (PI) was purchased from ApFfigplichem. Mouse monoclonal IgG1 antibody was purchased from Santa Cruz Biotechnology. Goat anti-mouse IgG Alexa 488 was purchased from Molecular Probes. Glutaraldehyde (25% acqueous solution) and Sodium Cacodylate (powder) were purchased from TAAB Laboratories Equipment Ltd. Osmium (VIII) oxide (OsO4) for microscopy was purchased from Merck KGaA. Polyethersulfone filter unit membranes were purchased from Termo Fisher Nalgene.

### Cell cultures

The human A549 adenocarcinoma alveolar basal epithelial cell line from the American Type Culture Collection (ATCC) was maintained in RPMI and the human HGF-1 from the American Type Culture Collection (ATCC) was maintained in DMEM respectively, Culture media were supplemented with 10% fetal bovine serum (FBS), 1% non-essential amino acids, 2 mM glutamine, 50 IU/ml penicillin and 50 µg/ml streptomycin (Complete Tissue Culture, CTC, medium). Cell cultures were maintained at 37 °C in a 5% CO2 humidified atmosphere in the air.

### Dispersions of NPs

An initial suspension of 1.7 g/ml ZnO NPs in H_2_O (Sigma Aldrich) was diluted in Milli Q water to a solution of 1 g/ml and sonicated using a sonicator equipped with a 6.5 mm probe for 45 min (Vibracell 75,041 ultrasonifier, Sonics & Materials Inc, 750 W, 20 kHz, 20% amplitude) under controlled temperature conditions.

For cytotoxicity tests, to mimic the biological milieu, a suspension protocol has been developed in culture media(Additional file 6). NPs suspensions in MilliQ water were diluted in the Serum-Free Culture (SFC) medium containing 1% water and used in the cytotoxicity assays at the final concentrations of 1, 5 and 20 μg/cm^2^.

### Dynamic Light Scattering measurements

Dynamic Light Scattering (DLS) has been employed to determine the hydrodynamic size of the nanoparticles. A NanoZetaSizer (Malvern) apparatus equipped with a 5 mWHeNe laser and with the thermostated cell was used. Samples were prepared as described in section “Dispersion of NPs” This instrument employs a backscatter detection, i.e. the scattered light is collected at an angle of 173°, this detection geometry gives the advantage to be less sensitive to multiple scattering effects concerning the more conventional collection angle of 90°. The measured autocorrelation functions were analyzed in the framework of the Mie theory by using cumulant method and the NNLS algorithm to get the number size distribution of samples [64].

### Transmission electron microscopy

For transmission electron microscopy observations, NPs suspensions (50 µg/ml) were differently diluted (1:10, 1:100 and 1:1000) and transferred onto prepared carbon-coated copper grids (Cu, hexagon, 300 mesh), allowed to dry and directly observed by TEM, using a Philips EM208 microscope, operating at an accelerating voltage of 80 kV.

### Variable pressure scanning electron microscopy (VP-SEM)

For VP-SEM observations, ZnO NPs suspensions (1, 5, and 20 µg/cm^2^) were transferred onto a polyethersulfone filter unit membrane (Supor® machV, Termo Fisher Nalgene®, USA), allowed to dry and directly observed by a Hitachi SU 3500 Scanning electron microscope (Hitachi High-Technologies Europe GmbH, Mannheim, Germany), operating at 4 kV, the working distance of 4.9 mm, and 40 Pa, in BSE COMPO mode without metal coating. This particular innovative microscope is equipped with several detectors: secondary electrons detector (SE), ultravariable-pressure detector (UVD) and backscattered electrons detector (BSE). This instrument allows NPs observation at variable pressure, humidity and low voltage, avoiding sample physical damage due to the effect of the high voltage electron beam and high vacuum condition. UVD detects photons generated by the interaction of accelerated secondary electrons with gas molecules in the low vacuum environment of the chamber, so it is particularly suitable to obtain surface information at low vacuum and low accelerating voltages. BSE image COMPO mode in a low vacuum means that the image results from the overlapping of images generated by secondary electrons ad backscattered electrons.

### Treatments

For treatments with ZnO nanoparticles, after 24 h from seeding, CTC medium was substituted with RPMI supplemented with 1% non-essential amino acids, 2 mM glutamine, 50 IU/ml penicillin and 50 µg/ml streptomycin, 1% water without FBS (Serum-Free Culture, SFC, medium). In all the experiments cells were treated with ZnO NPs (1, 5 and 20 μg/cm^2^), for 24, 48, 72 and 144 h. We used 1 μM Staurosporine (STS, Sigma-Aldrich, Saint Lois, Missouri, USA) as positive control of apoptosis induction and 10 μM Rapamycin (Rap, Sigma-Aldrich) as an autophagic positive control for 24, 48, 72 and 144 h.

### MTT and Neutral Red (NR) assays

To maintain NPs/surface and NPs/cells identical to that of ECIS test, A549 cells were seeded in 96-well plates at the density of 28,880 cells/well in CTC medium (200 μl).

After 24 h, the CTC medium was substituted with SFC medium and tumour cells were treated as described in the "Treatments" section.

For MTT Assay, after removing cell medium, cells were washed with PBS and incubated with 0.5 mg/ml MTT, (3-[4,5-dimethylthiazol-2-yl]-2,5-diphenyl-tetrazolium bromide solution (Sigma) for 2 h at 37 °C. After removing the MTT solution the samples were lysed by 100 μl DMSO and analysed by a microplate reader (BioRad, California) at 570 nm. Cell viability (%) was calculated as follows: (absorbance mean value of the treated sample/absorbance mean value of the control sample) × 100, after normalizing the absorbance of each well to the absorbance value of the blank [65].

For NRU assay, we followed the protocol according to [31]. Briefly, after removing cell medium, cells were washed with PBS and incubated with 100 μl of 40 µg/ml neutral red (3-amino-7-dimethylamino-2-methyl-phenazine hydrochloride) dye for 2 h at 37 °C. After removing Neutral red dye solution the cells were washed with PBS and incubated with 150 μl Neutral red destain solution (50% ethanol 96%, 49% deionized water, 1% glacial acetic acid) for 10 min at 37 °C. Finally, 96-well plates were analysed by a microplate reader (BioRad, California) at 540 nm. Cell viability (%) is calculated as follows: (absorbance mean value of the treated sample/absorbance mean value of the control sample) × 100, after normalizing the absorbance of each well to the absorbance value of the blank.

### Electric cell-substrate impedance sensing

Electric cell-substrate impedance sensing (ECIS) measurements were performed at the frequency f = 4000 Hz, which was found to yield the maximum change in resistance of the cell-covered electrode [30]. Resistance and capacitance were normalised at respective values for cell-covered electrodes, immediately before treatment.

At first, two new 8W20idf PET arrays with 8 empty wells each were connected to the ECIS system. Then the arrays were placed in an incubator at 37 °C, with 5% CO2, allowing for a sufficient time to obtain the desorption of chemical substances, possibly present on the surface of the wells’ electrodes, as recommended by the manufacturer. A549 cells were seeded on the 8WCP + PET array’s wells, at the density of 40.000 cells/well in CTC medium (500 μl). After 24 h (following [66], CTC medium was substituted with SFC medium and A549 cells were treated with ZnO NPs (1, 5 and 20 μg/cm^2^), and continuously monitored for at least 144 h. The treatments were carried out in a laminar-flow biological hood. As a positive control we used 1 μM Staurosporine (STS, Sigma) or 10 μM Rapamicine (Rap, Sigma), both in the same array of the treated cells, and continuously monitored for at least 144 h. Another series of experiments addressed the behaviour of another tissue type (HGF-1 cell line, gingival fibroblasts) with the same treatments delivered to A549 cells. Details of the procedure are available in the Additional file 6.

### Flow cytometry analysis

#### Apoptosis analysis

Flow cytometry was used to measure Annexin V-fluorescein isothiocyanate (FITC)/PI staining to investigate the mode of cell death induced by ZnO NPs. Annexin V-FITC/PI staining was carried out with an Annexin V-FITC apoptosis detection kit (BD Bioscience, Becton, Dickinson & Company). Briefly, A549 cells were plated in 6-well at the density of 5*10^5^ cells/well in CTC medium. After 24 h, CTC medium was substituted with SFC medium and tumour cells were treated as previously described. After treatments, cells were detached, centrifuged and resuspended in binding buffer 1X. The cell suspension was then incubated with 5 µl of Annexin V-FITC solution for 15 min. After washing with binding buffer 1X, cells were incubated with 5 µl of PI and immediately analyzed by flow cytometer.

#### Bivariate analysis of DNA content versus cyclin B expression

Untreated and treated A549 cells were collected, washed with cold PBS and suspended in 80% ethanol at – 20 °C for 2 h. The samples were then centrifuged, washed with PBS, and treated with 0.25% Triton X-100 in PBS for 5 min on ice. After washing with PBS, samples were incubated with mouse monoclonal IgG1 antibody (Santa Cruz Biotechnology, Inc., Dallas, USA) diluted 1:100 in PBS containing 1% BSA for 30 min. Cells were then washed and incubated with goat anti-mouse IgG Alexa 488 (Molecular Probes, USA) diluted 1:50 in PBS plus 1% BSA for 20 min. Then cells were washed again, resuspended in PBS containing 100 μg/ml Ribonuclease A (RNAse) and 40 μg/ml PI at room temperature for 20 min before flow cytometric analysis [67]. As isotypic control, we used an isotype-specific antibody (Sigma).

The fluorescent signals were analyzed by a BDLSRII flow cytometer (Becton, Dickinson & Company, Franklin Lakes, NJ, USA) equipped with a 5 mW, 488 nm, air-cooled argon-ion laser and a KimmonHeCd 325 nm laser. The fluorescence emissions were collected through a 530-nm bandpass filter for fluorescein isothiocyanate conjugated antibody (FITC), a 575-nm bandpass filter for propidium iodide (PI). At least 10,000 events/sample were acquired in linear mode for cell cycle and cyclin B expression studies and log mode for Annexin V-FITC labelling. Percentages of cells in subG1, G1, S and G2/M phases of the cell cycle, cyclin B expression, and percentages of early and late apoptotic cells were calculated using the FACS Diva software (Becton, Dickinson & Company).

### Cloning efficiency

The clonogenic survival test was used to determine the cell sensitivity to ZnO NPs. Briefly, 15*10^4^ cells were seeded in triplicate in 24 multiwell plates. After 24 h, cultures were treated as described in "Treatments" section, incubated at 37 °C for 24, 48, 72 and 144 h. Then, cells were detached, plated (1000 cells per 60 mm tissue culture dish) and allowed to grow for 10–14 days. After growth, cell colonies were fixed with 95% ethanol, for 15 min, and stained with a methylene blue solution in 80% ethanol, for 2 h. Only colonies composed of more than 50 cells were evaluated. The surviving fraction was calculated by dividing the colony number of treated cells by the number of colony number of untreated cells. Data represent the mean of three independent experiments.

### Immunostaining

Immunostaining for fluorescence microscopy was used to study the alteration of cytoskeleton and nuclei upon ZnO NPs exposure. At the end of treatments, samples were washed with PBS and fixed in 3.7% formaldehyde for 15 min. After removing formaldehyde, samples were washed twice in PBS, treated with 0.5% Triton X-100 for 5 min, and blocked in blocking solution (10% FBS, 1% bovine serum albumin in PBS) for 30 min. Microtubule staining was performed by using mouse monoclonal anti-α/β tubulin antibody mixture (Sigma Biosciences, Italy) diluted 1:50 in PBS with 1% BSA, followed by a mixture of secondary Alexa 488 goat anti-mouse IgG (1:50; Molecular Probes, USA) and phalloidin TRITC (1:100) to detect f-actin. Nucleus staining was carried out with PI (40 µg/ml) in PBS for 15 min. Cells were rinsed three times with PBS afterwards. Finally, samples were mounted in PBS containing 50% glycerol. Observations were performed by a Leica TCS SP2 laser scanning confocal microscopy (Leica, Microsystems, Mannheim, Germany) equipped with an Ar/Kr laser.

### Scanning electron microscopy

For scanning electron microscopy (SEM) analysis, cells treated with ZnO NPs, at the indicated times, were fixed with 2% glutaraldehyde in 0.1 M cacodylate buffer (pH 7.4) at room temperature for 30 min, post-fixed with 1% OsO_4_ in the same buffer, dehydrated through a graded ethanol series, critical point dried with CO2 (CPD 030 Balzers device, Bal-Tec, Balzers), and gold-coated by sputtering (SCD040 Balzers device, Bal-Tec). Samples were examined with a field emission gun scanning electron microscope (SEM-FEG, Quanta 200 Inspect, FEI Company, Eindhoven, The Netherlands).

## Supplementary Information

**Additional file 1: **Figure S1. Characterization of ZnO-NPs. **a-d** Images of ZnO-NPs observed at VP-SEM in BSE-COMPO mode, without metal coating. Magnification: 8.000X, dotted bar: 5 µm. **a **Low vacuum secondary electron mode (UVD). **b **Backscattered electron in composition mode (BSE-COMP). **c **Backscattered electron in topography mode (BSE-TOPO). **d **image in COMPO mode, obtained by integrating **a**, **b **and **c**, images to get an improved contrast and quality and facilitates the identification of the NPs. e DLS number weighted distribution of hydrodynamic diameter of ZnO NPs obtained by NNLS algorithm. Solid line represents the Gaussian fit used to determine the mean and the width of the size distribution. DLS data obtained by cumulant analysis (the mean size and polydispersity of the sample, i.e. Z- average and PDI, respectively) and by NNLS number weighted analysis (mean diameter and distribution width) are reported in the table. Errors correspond to the standard deviations of three repeated measurements. **f **TEM image analysis and particle size histogram.

**Additional file 2: Figure S2. **Effects of 1, 5 and 20 mg/cm2 ZnO-NPs on A549 cell survival Trypan blue assay (TB). A549 cells were treated with ZnO-NPs (1, 5 and 20 μg/cm2), Rap (10 μM Rapamycin) and STS (1 μM Staurosporine) for **a **24, **b **48, **c **72, and **d **144h. TB assay confirmed that, after ZnO-NPs exposure, the survival rate of A549 cells underwent substantial decreases in a time and dose-dependent manner. Experiments were performed in triplicate. Error bars represent SD.

**Additional file 3: Figure S3. **Effect of ZnO-NPs on cell cycle of A549 cells. The effect on cell cycle phases was clearly evident after treatment with the highest dose of ZnO-NPs (20 mg/cm2). STS (1 μM Staurosporine) was used as positive control of apoptosis induction. The percentage of cells in G2/M phase noticeably increased in cultures treated for 24, 48 and 72 h. With regard to the other cell cycle phases (subG1, G1 and S) no relevant alterations were revealed.

**Additional file 4: Figure S4. **Interaction of ZnO-NPs with HGF-1 cells. Scanning electron microscopy observations. **a **HGF-1 cells treated with 5 µg/cm2 ZnO-NPs for 24 h. **b-d** HGF-1 cells treated with 20 µg/cm2 ZnO-NPs for 24 h. ZnO-NPs NPs (arrows) interact with surface and fibrous matter of HGF-1 cells. 

**Additional file 5: Figure S5. **A549 cells treated with ZnO-NPs. Observations performed by scanning electron microscopy. The treatment with ZnO-NPs induced time- and dose-dependent morphological changes in A549 cell cultures. Cultures treated with low doses of ZnO-NPs for 24 h displayed several mitotic cells in comparison with control cultures (arrows). Numerous cells rounded in shape were visible in cultures treated with 5 µg/cm2 ZnO-NPs for 48 h suggesting a dramatic loss of adhesive properties of A549 cells. A recovery was visible at 72 h. The exposure to 20 µg/cm2 ZnO-NPs induced dramatic modifications of the morphology and cell detachment, suggesting strong alteration of the cytoskeletal structure.

**Additional file 6:** SOP experimental procedures.

## Data Availability

All data are available in the main manuscript and additional file.

## Abbreviations

ATCC: American type culture collection
BSA: Bovine serum albumin
BSE: Backscattered electrons detector
CPD: Critical point dried with CO2
CTC: Complete tissue culture
DMSO: Dimethyl sulfoxide)
ECIS: Electric cell-substrate impedance sensing
FBS: Fetal bovine serum
DLS: Dynamic light scattering
LDH: Lactate dehydrogenase
MTT: 3-[4,5-Dimethylthiazol-2-yl]-2,5-diphenyl-tetrazolium bromide solution
NPs: Nanoparticles
NR: Neutral red
PBS: Phosphate-buffered solution
PI: Propidium iodide
Rap: Rapamycin
ROS: Reactive oxygen species
RNAse: Ribonuclease A
SE: Secondary electrons
SEM-FEG: Field emission gun scanning electron microscope
SFC: Serum-free culture
STS: Staurosporine
TB: Trypan blue
TEM: Transmission electron microscopy
UVD: Ultravariable-pressure detector
VP-SEM: Variable pressure scanning electron microscopy
WST1: Water soluble tetrazolium-1
