# Supplementary figures and images for: Label-free cell based impedance measurements of ZnO nanoparticles—human lung cell interaction: a comparison with MTT, NR, Trypan blue and cloning efficiency assays
Source: J Nanobiotechnology. 2021 Oct 7;19:306. doi: 10.1186/s12951-021-01033-w (PMC8499537; doi:10.1186/s12951-021-01033-w)

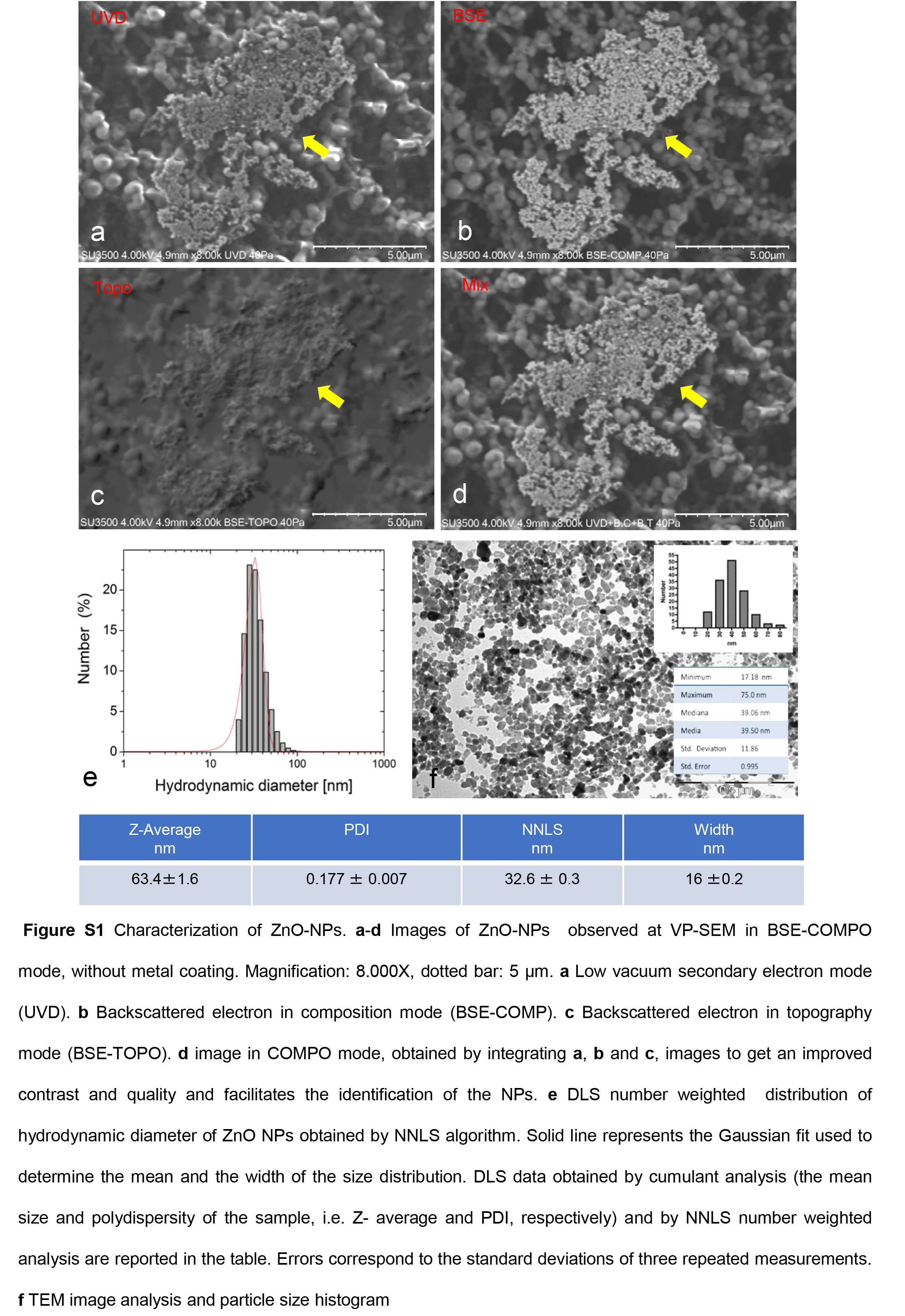

Supplement: Supplementary file 1 — Additional file 1: Figure S1. Characterization of ZnO-NPs. a-d Images of ZnO-NPs observed at VP-SEM in BSE-COMPO mode, without metal coating. Magnification: 8.000X, dotted bar: 5 µm. a Low vacuum secondary electron mode (UVD). b Backscattered electron in composition mode (BSE-COMP). c Backscattered electron in topography mode (BSE-TOPO). d image in COMPO mode, obtained by integrating a, b and c, images to get an improved contrast and quality and facilitates the identification of the NPs. e DLS number weighted distribution of hydrodynamic diameter of ZnO NPs obtained by NNLS algorithm. Solid line represents the Gaussian fit used to determine the mean and the width of the size distribution. DLS data obtained by cumulant analysis (the mean size and polydispersity of the sample, i.e. Z- average and PDI, respectively) and by NNLS number weighted analysis (mean diameter and distribution width) are reported in the table. Errors correspond to the standard deviations of three repeated measurements. f TEM image analysis and particle size histogram. [file 12951_2021_1033_MOESM1_ESM.tif]

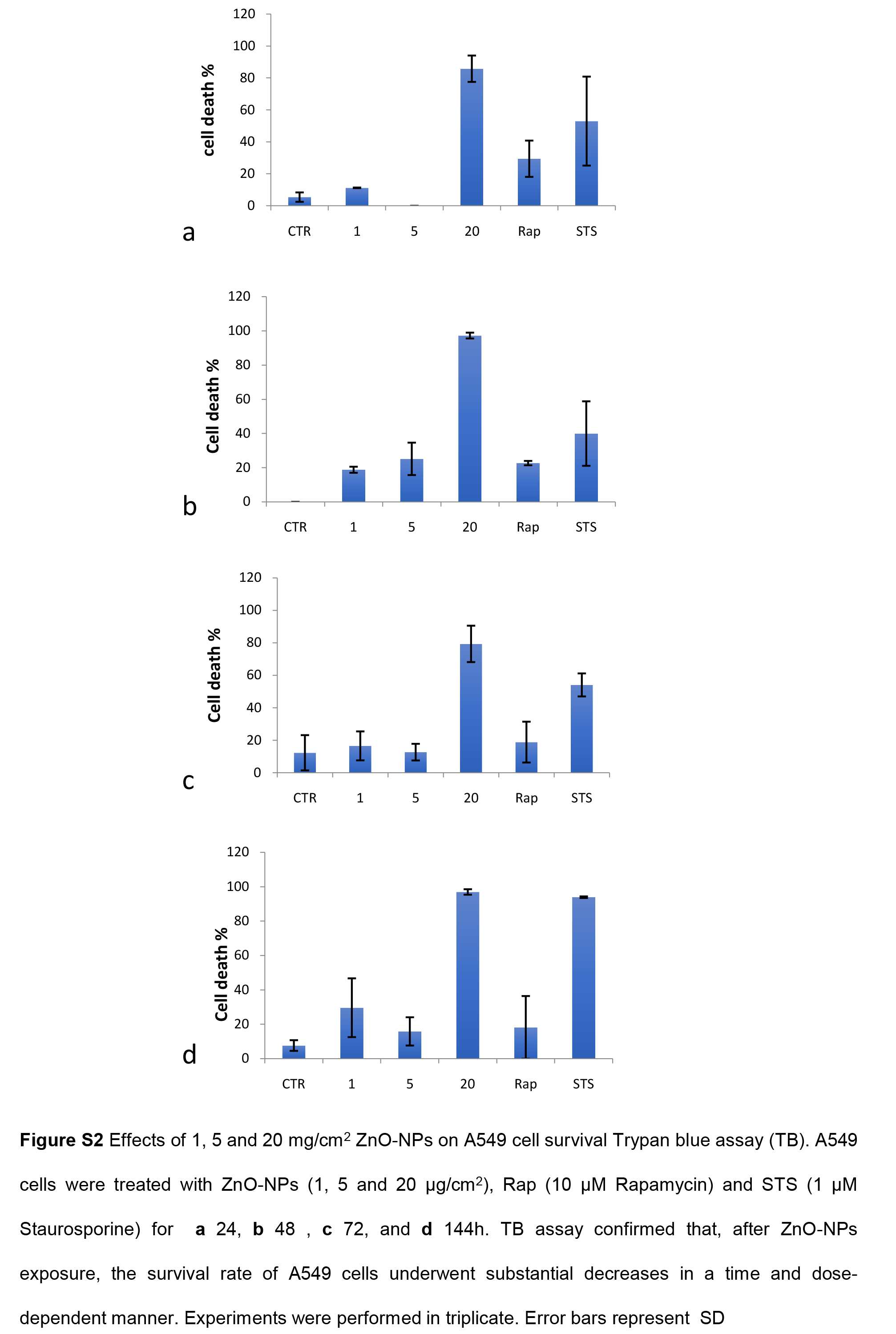

Supplement: Supplementary file 2 — Additional file 2: Figure S2. Effects of 1, 5 and 20 mg/cm2 ZnO-NPs on A549 cell survival Trypan blue assay (TB). A549 cells were treated with ZnO-NPs (1, 5 and 20 μg/cm2), Rap (10 μM Rapamycin) and STS (1 μM Staurosporine) for a 24, b 48, c 72, and d 144h. TB assay confirmed that, after ZnO-NPs exposure, the survival rate of A549 cells underwent substantial decreases in a time and dose-dependent manner. Experiments were performed in triplicate. Error bars represent SD. [file 12951_2021_1033_MOESM2_ESM.tif]

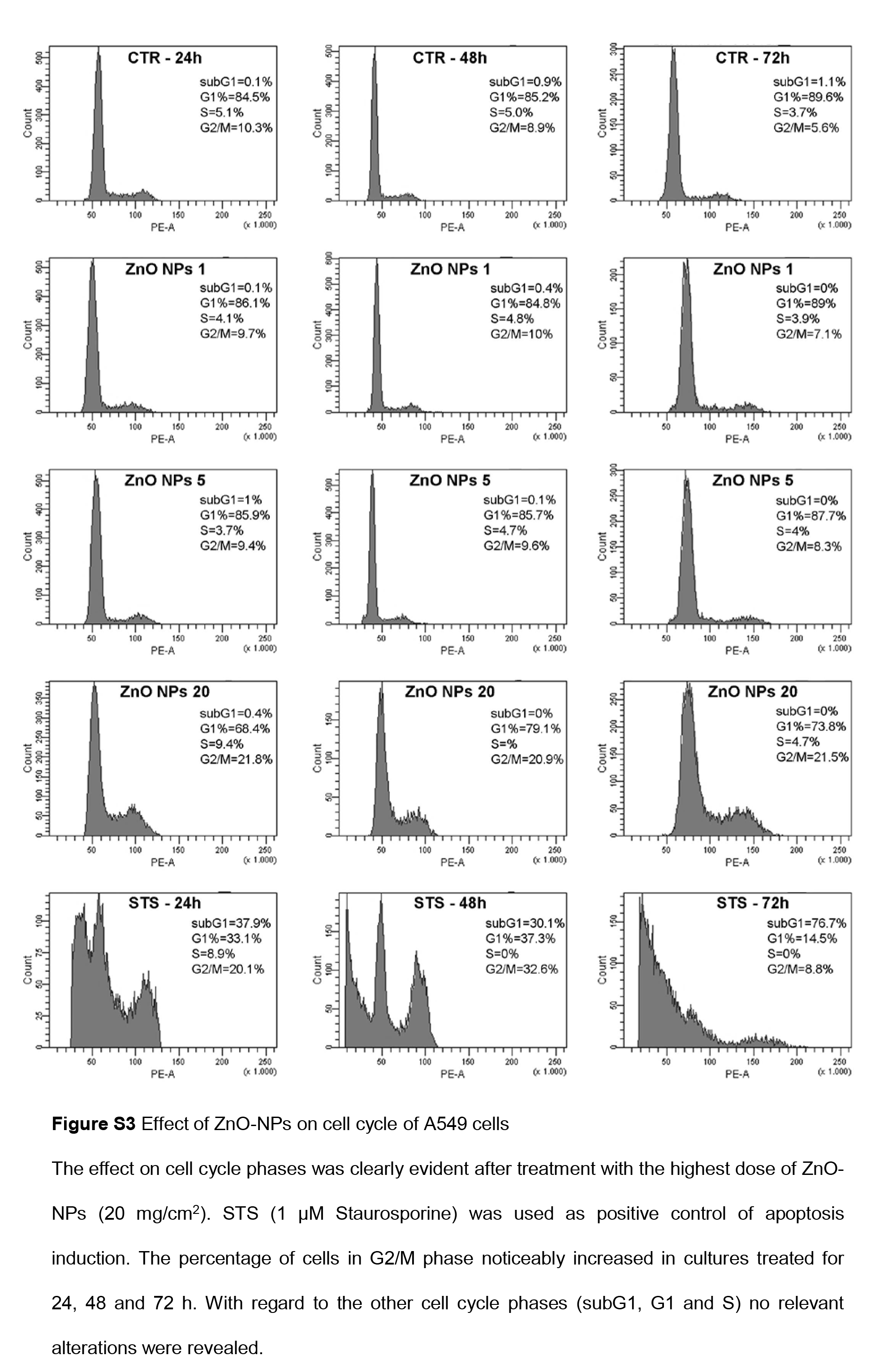

Supplement: Supplementary file 3 — Additional file 3: Figure S3. Effect of ZnO-NPs on cell cycle of A549 cells. The effect on cell cycle phases was clearly evident after treatment with the highest dose of ZnO-NPs (20 mg/cm2). STS (1 μM Staurosporine) was used as positive control of apoptosis induction. The percentage of cells in G2/M phase noticeably increased in cultures treated for 24, 48 and 72 h. With regard to the other cell cycle phases (subG1, G1 and S) no relevant alterations were revealed. [file 12951_2021_1033_MOESM3_ESM.tif]

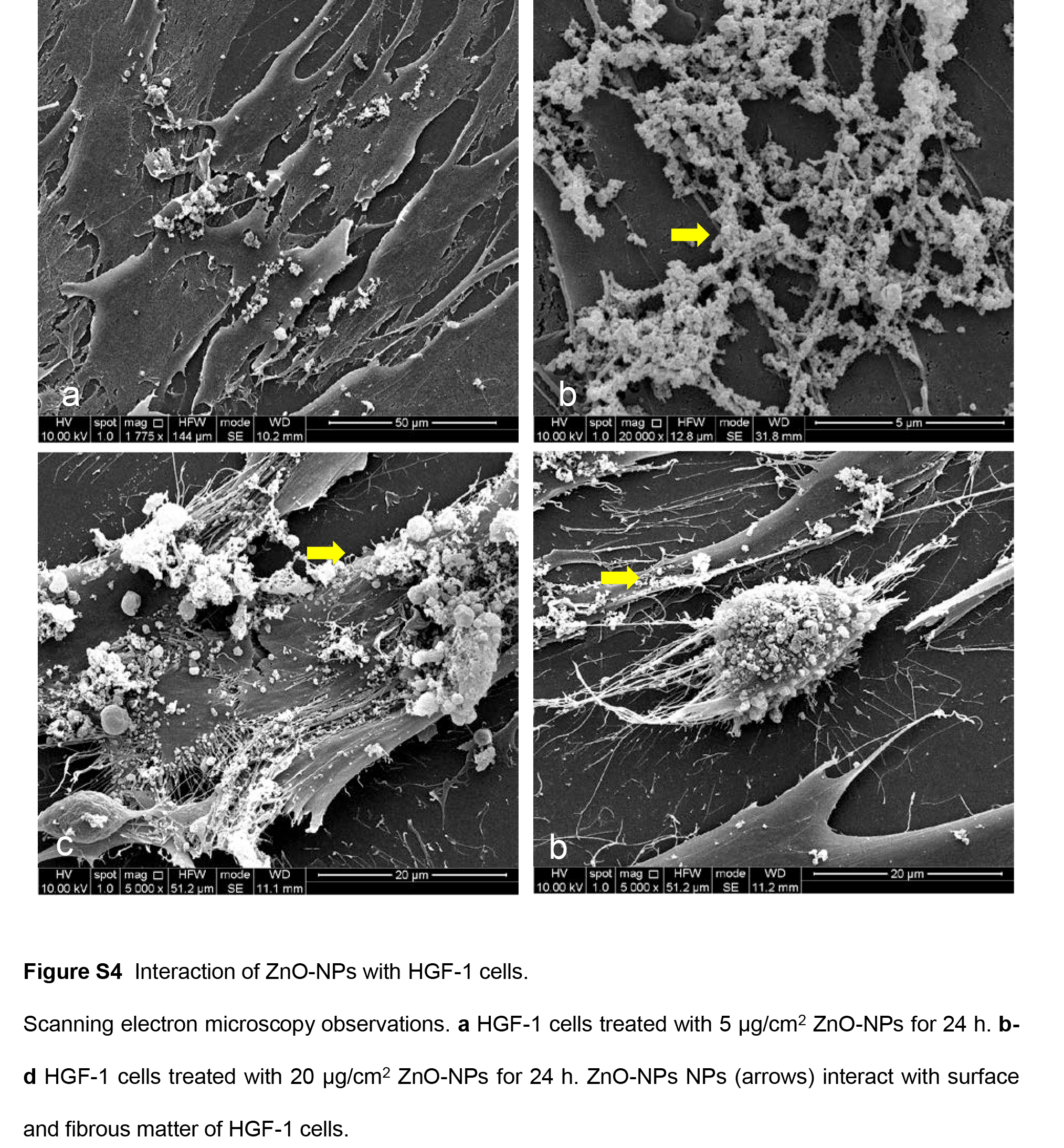

Supplement: Supplementary file 4 — Additional file 4: Figure S4. Interaction of ZnO-NPs with HGF-1 cells. Scanning electron microscopy observations. a HGF-1 cells treated with 5 µg/cm2 ZnO-NPs for 24 h. b-d HGF-1 cells treated with 20 µg/cm2 ZnO-NPs for 24 h. ZnO-NPs NPs (arrows) interact with surface and fibrous matter of HGF-1 cells. [file 12951_2021_1033_MOESM4_ESM.tif]

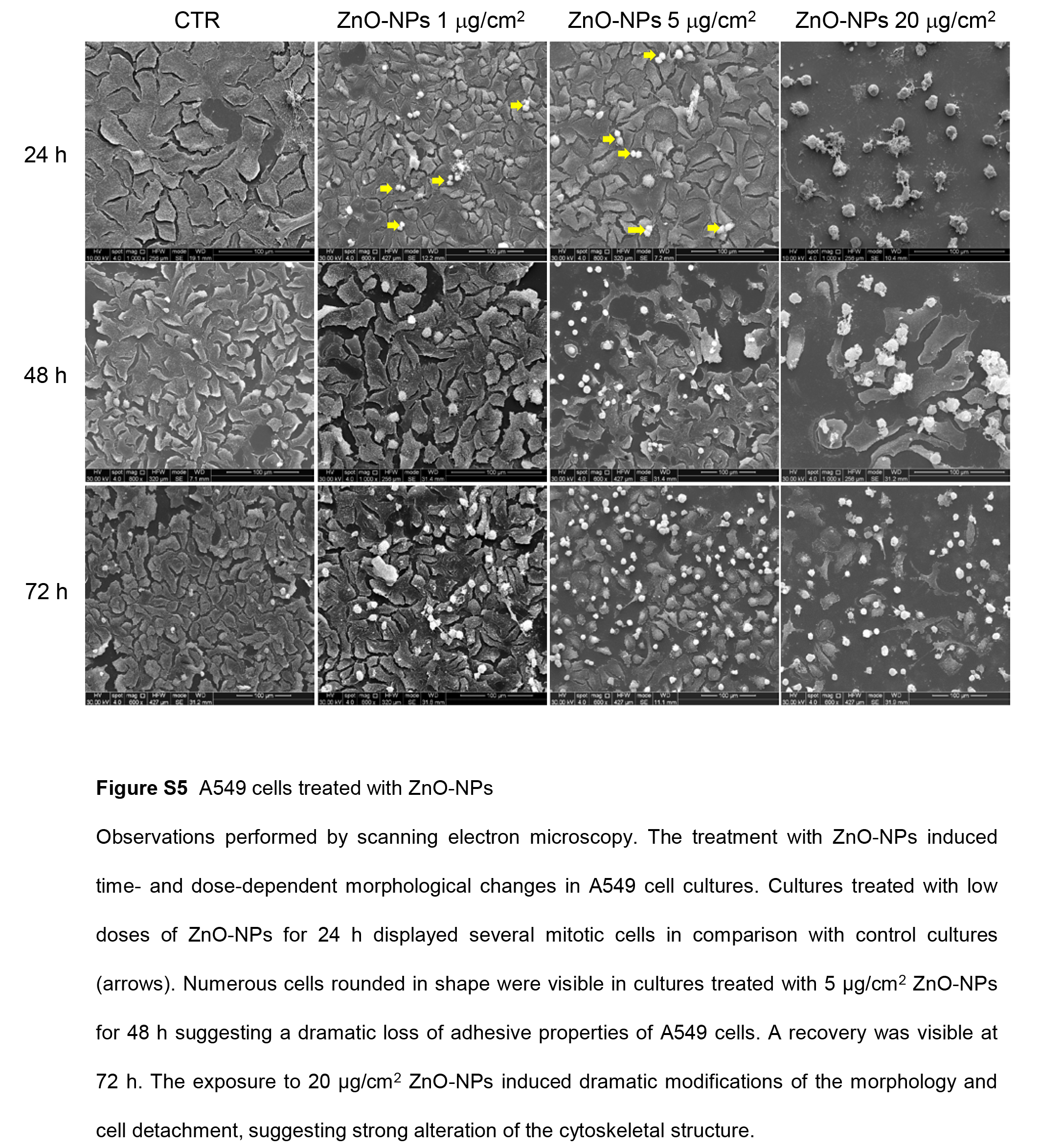

Supplement: Supplementary file 5 — Additional file 5: Figure S5. A549 cells treated with ZnO-NPs. Observations performed by scanning electron microscopy. The treatment with ZnO-NPs induced time- and dose-dependent morphological changes in A549 cell cultures. Cultures treated with low doses of ZnO-NPs for 24 h displayed several mitotic cells in comparison with control cultures (arrows). Numerous cells rounded in shape were visible in cultures treated with 5 µg/cm2 ZnO-NPs for 48 h suggesting a dramatic loss of adhesive properties of A549 cells. A recovery was visible at 72 h. The exposure to 20 µg/cm2 ZnO-NPs induced dramatic modifications of the morphology and cell detachment, suggesting strong alteration of the cytoskeletal structure. [file 12951_2021_1033_MOESM5_ESM.tif]
