# Supplementary material for: Label-free cell based impedance measurements of ZnO nanoparticles—human lung cell interaction: a comparison with MTT, NR, Trypan blue and cloning efficiency assays
Source: J Nanobiotechnology. 2021 Oct 7;19:306. doi: 10.1186/s12951-021-01033-w (PMC8499537; doi:10.1186/s12951-021-01033-w)
Supplement: Supplementary file 6 — Additional file 6: SOP experimental procedures. [file 12951_2021_1033_MOESM6_ESM.docx]

“**Electric cell-substrate impedance sensing to analyze the interaction of ZnO nanoparticles with human tumour (A549) and normal (HGF-1) cell lines: a comparison with endpoint measurements of multiple independent assays**” *by G. Bozzuto, G. D’Avenio et al*

This SOP was developed in the context of the Agreement (Ministry of Health – N. 9330) between the Istituto Superiore di Sanità and the Ministry of Health. The Agreement concerns the technical-scientific support to the Ministry of Health for the evaluation of the safety of Medical Devices subject to clinical investigations as defined by the art. 14 of Legislative Decree 46/97 and by art. 7 of the legislative decree 507/92 as modified by the legislative decree n. 37 of 2010.

The need for investigations on MDs carrying “nanostructures” is found in the recent amendments and additions to the Community Directives of reference in the sector, which have introduced the need for specific risk analysis in concerning these materials for all medical devices carrying nanostructures. In fact, the medical device market is characterized by devices that feature increasingly innovative technologies, a front of which the need for a risk assessment, according to specific regulations, for their safe use is evident.

The cytotoxicity tests contemplated in the EN ISO 10993-5 standards were developed and compared with a cytotoxicity test based on measurement (Electric Cell-substrate Impedance Sensing, ECIS) as a complement to the experimental procedures aimed at the biological evaluation of DM carrying nanostructures.

In the hope that it will be useful for the readers, the materials and methods, and the procedures employed in the different experimentation phases are herein reported. Some critical issues, and suggestions to overcome it were reported in blue characters.

**Electric Cell-substrate Impedance Sensing**

Electric Cell-substrate Impedance Sensing (ECIS) is a non-invasive method, by which physiological and morphological aspects of a cell population (in static or dynamic culture) can be monitored in real-timekinetic. The instrument, in fact, measures the impedance variations (in its two components capacitance andresistance) of the interface between the electrode at the bottom of each well of the plate, and the electrolyte represented by the culture medium. Thus the culture in the well is represented by a simple and interpretable electronic network model, given the known electrochemical knowledge of the cell populations and the culture medium used, together with biochemical knowledge a priori determined by the good laboratory practices, already tested.

For the model used by ECIS, it must be said that when the inoculated cells stick to the bottom of the well and begin to form the monolayer, essentially behave as a layer of insulating, thus inducing a non-linear decrease in capacity, and a linear increase in resistance, when compared to a well with culture medium alone. The cellular characteristic underlying this type of experiments is that it is due to the cell response to an external stimulus(drugs, physical agents, binding to a ligand, interaction with NPs).Cells respond by changing their morphology and consequently their interactions with the substrate to which they are associated, with the consequent change in impedance recorded by the electrode. Through ECIS it is possible to correlate these changes with the corresponding cellular event, possibly with the help of microscopy recordings.

By ECIS it is possible to perform measurements at different excitation frequencies of the preparation a starting from 0.4 kHz up to 40 kHz. The selection of the best excitation frequency depends on the particular experiment designed, and it is possible to determine this frequency by performing some preliminary evaluations. For most traditional experiments this frequency is that of 4kHz, also found in the literature. Measurements with ECIS can be used to monitor cell proliferation. Alterations in the culture conditions can in fact affect the speed with which the cell monolayer reaches confluence.

**Set up of ECIS system and comparison with MTT test**

For the development of the ECIS system and for the comparison with cytotoxicity tests we used the human alveolar adenocarcinoma A549 cell line, frequently employed as airway epithelium model in NP cytotoxicity tests (TiO2, ZnO etc.).

In a first experimental phase, we compared the ECIS test with the viability/cytotoxicity test was developed and compared cellular based on MTT (tetrazolium salt, [3- (4,5-dimethylthiazol-2-yl) -2,5-diphenyltetrazoliumbromide]).

**CELL VITALITY / CYTOXICITY TEST USING MTT (PHASES M1-M7)**

*PHASE M1 - Preparation of cell culture in flat-bottom 96-well plates*

All phases were carried out in a laminar flow biohazard cabinet.

M1.1 Preparation of complete tissue culture medium

The culture medium used was the RPMI-1640 added with L-glutamine, penicillin (50 IU/ml),streptomycin (50 μg/ml), 10% FCS (complete tissue culture medium, CTC). The medium was stored a +4°C.

Note: The addition of antibiotics to the medium is recommended for NP exposure experiments. We also recommend the calibration of the cell incubator for temperature (37°C), humidity (95%), and concentration of CO_2_(5%).

M1.2 Preparation of single-cell suspension

The single-cell suspension was prepared as follows:

- CTC, EDTA and trypsin were pre-heated to 37 °C in a thermostated water bath.

- The cell cultures were washed with an EDTA solution (10 mM, 0.5 ml for T25culture flasks), by shaking lightly for a few seconds.

- A trypsin solution (0.25%, 0.5 ml for T25 culture plates) was added by shaking lightly for a few seconds.

- The cultures were then incubated in a humidified CO_2_ incubator at 37 °C, for 3-5 min.

- After checking the culture by inverted light microscope to see if cells get rounded and detached from the bottom of the flask, 5 ml of CTC was added, gently pipetting cells to disperse them homogeneously.

Note: it is particularly important to obtain a single-cell suspension to make an appropriate cell count.

M1.3 Count of cells contained in the suspension

The cell count was performed in the following ways:

- 20 μl of cell suspension obtained in phase M1.2 was transferred with a micropipette ina sterile Eppendorf tube.

- After the addition of 20 μl of a Trypan bluesolution (TB) (dilution factor 1: 2),10 μl of the suspension were taken and loaded into the cell counting chamber (Neubauer chamber).

- The cells were then counted under the light microscope in the four quadrants of the chamber. The count was repeated twice.

The calculation of the number of viable cells (TB negative) was carried out by applying the following

formula:N = {[(a+b)/8]*10.000*DF}

where:

N = number of cells/ml

a = number of cells counted in the 4 quadrants of the first chamber

b = number of cells counted in the 4 quadrants of the second chamber

8 = number of quadrants counted x10,000 (10^4^) = conversion factor for the chamber volume

DF = dilution factor of the cell suspension (generally equal to 2);

- The final volume of suspension was then calculated to obtain these eding density(cell/ml) to be used (M1.4).

M1.4 Cell seeding

For comparison with the data generated by the ECIS 8W20idf PET arrays, 28,880 cells were seeded in eachwell of 96 flat-bottom plates, to maintain the ratio NP/surface and cell/surface identical to that calculated for the assays with ECIS (Phase E1.4).

- The cell suspension was first transferred into a V-shaped bin and then 200 μl of cell suspension were seeded into a flat-bottom 96-well plate by using a multichannel pipette according to the diagram shown in Figure 3.

Note: to optimize the homogeneity in the preparation, it is important to mix the cell suspension. This minimizes sedimentation of cells at the bottom of the tank.

- After settling under laminar flow at room temperature for 15 min, the plate was gently transferred into a humidified CO_2_incubator at 37 °C.

*PHASE M2 - Preparation of NP dilutions in the flat-bottom dosing-plate*

This procedure was performed in a laminar flow biohazard cabinet to minimize the contamination with bacteria or fungi. NPs standard solutions used in the tests come from Sigma Aldrich: they are solutions containing 1.7 g/ml of NPs in H_2_O, stored at 25 °C. In this phase it was decided to carry out the experiment in serum-free medium (SFS) instead of in CTC, as from current literature data the serum tends to create a coating on NPs (“protein corona” effect).

- The medium was then prepared without adding 10% FBS (SFS), but containing 1% H_2_O in a 50 ml tube (49.5 ml SFS + 0.5 ml sterile H_2_O), and it was mixed by inversion. The SFS medium (with added antibiotics) containing 1% H_2_O was used as a vehicle to dissolve the NPs.

- Under sterile conditions 2 ml of NP stock solution was added in a 15 ml polypropylene tube.

- The tube containing the NP suspension was immersed inH_2_O and ice and sonicated for 30 min.

- The solution was then transferred to column 1 of the flat-bottom 96-well assay plate (Figure 1).

- NP dilutions were prepared immediately prior to cell treatment. The scheme containing the repeated columns of serial dilutions of the NPs, is shown in Table 1.


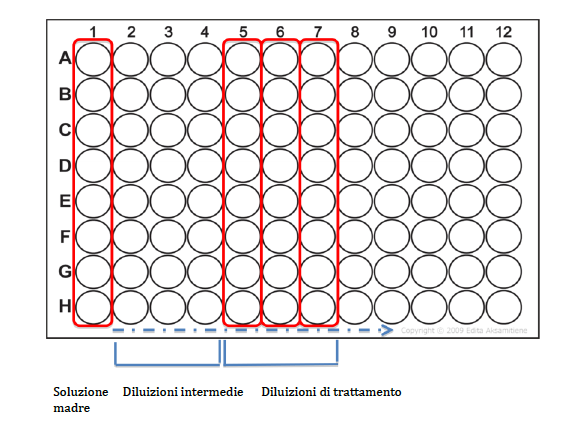


**Figure 1 - Flat-bottom 96-well dilution plate**

|  | Column  1 | Column  2 | Column  3 | Column  4 | Column  5 | Column  6 | Column  7 |
| --- | --- | --- | --- | --- | --- | --- | --- |
| A | 1g/ml | 20 mgtot  (1:10) | 2 mgtot  (1:10) | 0,2 mgtot  (1:10) | 20 μg tot  (1:10) | 5 μg tot  (1:4) | 1 μg tot  (1:5) |
| B |  | 20 μl Col 1  +180 μlSFS | 20 μl Col 2  +180 μlSFS | 20 μl Col 3  +180 μlSFS | 20 μl Col 4  +180 μlSFS | 50 μl Col 5  +150 μlSFS | 40 μl Col 6  +160 μlSFS |
| C |  | 20 μl Col 1  +180 μlSFS | 20 μl Col 2  +180 μlSFS | 20 μl Col 3  +180 μlSFS | 20 μl Col 4  +180 μlSFS | 50 μl Col 5  +150 μlSFS | 40 μl Col 6  +160 μlSFS |
| D |  | 20 μl Col 1  +180 μlSFS | 20 μl Col 2  +180 μlSFS | 20 μl Col 3  +180 μlSFS | 20 μl Col 4  +180 μlSFS | 50 μl Col 5  +150 μlSFS | 40 μl Col 6  +160 μlSFS |
| E |  | 20 μl Col 1  +180 μlSFS | 20 μl Col 2  +180 μlSFS | 20 μl Col 3  +180 μlSFS | 20 μl Col 4  +180 μlSFS | 50 μl Col 5  +150 μlSFS | 40 μl Col 6  +160 μlSFS |
| F |  | 20 μl Col 1  +180 μlSFS | 20 μl Col 2  +180 μlSFS | 20 μl Col 3  +180 μlSFS | 20 μl Col 4  +180 μlSFS | 50 μl Col 5  +150 μlSFS | 40 μl Col 6  +160 μlSFS |
| G |  | 20 μl Col 1  +180 μlSFS | 20 μl Col 2 +180 μlSFS | 20 μl Col 3 +180 μlSFS | 20 μl Col 4 +180 μlSFS | 50 μl Col 5  +150 μlSFS | 40 μl Col 6  +160 μlSFS |
| H |  | 20 μl Col 1  +180 μlSFS | 20 μl Col 2  +180 μlSFS | 20 μl Col 3  +180 μlSFS | 20 μl Col 4  +180 μlSFS | 50 μl Col 5  +150 μlSFS | 40 μl Col 6  +160 μlSFS |
|  | 1000μg/μl | 100 μg/μl | 10 μg/μl | 1 μg/μl | 0.1 μg/μl | 0.025 μg/μl | 0.005 μg/μl |

**Table 1 - Scheme containing serial dilutions of NPs**

*PHASE M3 - Treatment of cells with NPs, STS, and Rap.*

M3.1Cell-seeded plates (phase M1) were treated as follows:

- after incubation for 24 h in a humidifiedCO_2_incubator at 37 °C, the plate was observed with a phase contrast microscope to check cell adhesion, density and any contamination.

- The medium (200 μl) was removed and the SFS was added in a volume equal to 128 μl for treatments with nanoparticles and 200 μl for treatment with Staurosporine (STS) or Rapamycin (Rap).

- The 96-well plates for MTT were treated by taking 72 μl from columns 5, 6 and 7(Figure 1, Table 1). In particular, a total of 7.2μg of NP (column 5), 1.8 μg (column 6), and 0.36 μg (column 7) were collected to be administered on the surface of 0.36 cm^2^ of each well to obtain concentrations corresponding to 20 μg/cm^2^, 5μg/cm^2^and 1 μg/cm^2^.

M3.2 Treatment of cells with STS

The cells were also treated with STS (1 μM), as positive control for apoptosis.

-An aliquot was taken from a 1mM solution and administered directly to the wells to havea concentration of 1μM (0.2 μl in 200 μl, dilution 1:1000).

M3.3 Treatment of cells with Rap

The cells were also treated with Rap (10 μM), as positive control for autophagy.

-An aliquot was taken from a 10 mM solution and administered directly to thewells to have a concentration of 10 μM (0.2 μl in 200 μl, dilution 1:1000).

- The plates were treated for 24, 48, 72, and 144 h according to the scheme shown in Figure 2. Each sample was set up in quadruplicate.


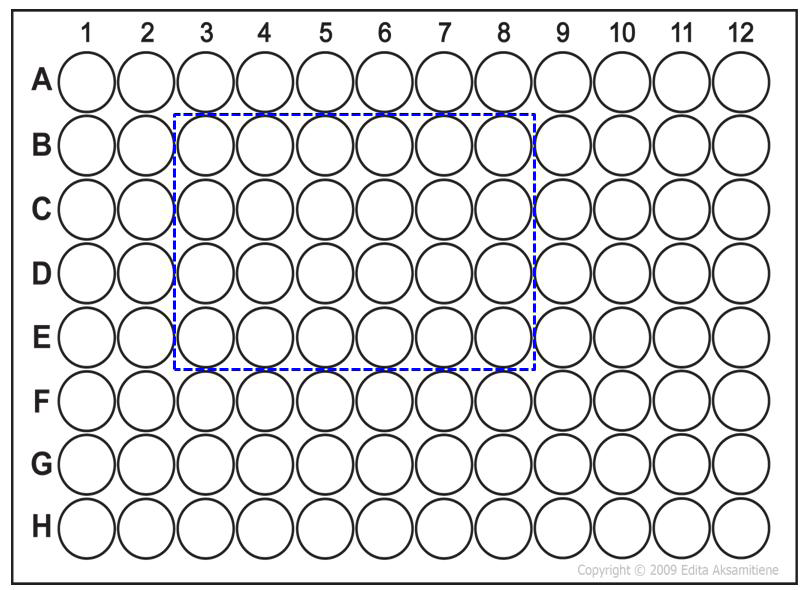


**Figure 2 - Flat-bottom 96-well treatment plate**

**Column 2:**blank

**Blue square**

**Column 3:** CTR

**Column 4:** ZnO20μg/cm^2^

**Column 5:** ZnO5μg/cm^2^

**Column 6:** ZnO1μg/cm^2^

**Column7:** Rapamycin 10 μM

**Column 8:** Staurosporine 1 μM

- At the end of the treatment, the cells were observed with a phase contrast microscope to verify the adhesion, and transferred into a humidified CO_2_ incubator for 24 h.

*PHASE M4 - Preparation of the solutions for the MTT test*

- 10 ml of MTT reagent were prepared, starting from a stock solution (5 mg/ml) by mixing 1 ml with 9 ml of PBS, to obtain a 0.5 mg/ml solution.

Note: For long-term storage, store the stock solution at -20 °C, protected from light.

*PHASE M5 - Check measuring equipment*

To verify the performance of the microplate reader, a flat-bottom 96-well plate was used.Each well were filled with equal volumes of reagent for the MTT assay.

The verification was carried out as follows:

- 10 ml of absorption solution was transferred into a V-shaped tank (the plastic containermust be sterilized before use).

- Then, by using a 6-channel multichannel pipette, 200 μl was added in eachwell of the plate to be read. The absorbance measurements of each well should not show obvious outliers.

*PHASE M6 - Execution of the MTT test*

- At the end of the NP exposure, the individual wells of the plate were observed at the microscope to check the morphology of the cells, their state of health, the percentage ofconfluence and possible contamination (in which case the plates should be discarded).

- Treatments and non-adherent cells were removed with a multichannel pipette.

- A wash with PBS (100 μl) was done.

- With a multichannel pipette with 8 tips, 100 μl of MTT reagent (0.5 mg/ml) wereadded in each well of the plate, avoiding the formation of air bubbles.

- The wells were checked for the presence of air bubbles and removed using a needlesyringe

Note: Air bubbles can significantly affect absorbance measurements made witha plate reader).

- The plate was incubated at 37 °C for 2 h in a humidified atmosphere at 5% CO2.

- The presence of blue precipitates in the cells was checked under a light microscope.

- The MTT reagent was then removed.

- 100 μl of DMSO were added to wells delimited by the blue square(control cells and cells treated with the various NP concentrations,andin column 11(blank).

Note: It is recommended to lysate the cells and dissolve the formazan crystals by pipetting each well.

- The plate was then incubated at 37 °C for 10 minutes.

- Finally, the absorbance at 570 nm was measured with a microplate reader.

Note: the amount of colored product is proportional to the number of live cells. If a treatmentcauses cell death, decreases absorbance.

*PHASE M7 - Data analysis*

The raw data of each well of the plate was recorded to calculate the final data and forthe analysis of the variation coefficient.

For the determination of cell viability it was then calculated as follows:

- the resulting absorbance from each well was normalized to the absorbance value of theblank (column 11).

- After normalization, it was calculated the average values ​​of each single treatment, carried out inquadrupled.

- The values ​​of the treated wells were then compared with the values ​​of the untreated wells. Valuesof the results represent the fraction of cells that survive after treatment and canbe expressed as a percentage of cell viability as the following formula:

% cell viability = [absorbance of treated cells / absorbance of untreated cells x 100].

- The standard deviation was finally determined.

**CYTOTOXICITY TEST USING ECIS TECHNOLOGY (ELECTRIC CELL – SUBSTRATE IMPEDANCE SENSING) (Phases E1-E3).**

For ECIS test 8WCP PET arrays, now 8W20idf PET, were used. Each array contains two sets (7.5mm x 0.5mm) of rectangular interdigitated electrodes. Each of the 8 wells has a total electrode area of 3.985mm^2^ located on inter-digitated fingers to provide measurements of cells. Each well has a substrate area of 0.8cm^2^ and a maximum volume of 600 μl.

Cell seeding and treatments were carried out in the arrays already connected to the ECIS device.

*PHASE E1 - Preparation of the cell culture in the ECIS arrays*

E1.1 Preparation of complete tissue culture medium (CTC).

E1.2 Preparation of the single cell suspension.

E1.3 Count of cells contained in the suspension.

The phases E1.1-E1.3 are equal to the phases M1-M3 described in the procedure of cell viability/cytotoxicity testby MTT.

E1.4 Cell seeding

-First, two new 8W20idf PET arrays, with 8 empty wells each, were connected to the ECIS system. Then the arrays were placed in an incubator at 37 °C, with 5% CO2, allowing for a sufficient time to obtain the desorption of chemical substances, possibly present on the surface of the well’s electrodes, as recommended by the manufacturer.

- A549 cells were seeded on the array’s wells, at the density of 40.000 cells/well in CTC medium (500 μl).

*PHASE E2 - Preparations of NP dilutions in the flat bottom assay plate*

- The necessary dilutions were prepared with SFS + 1% H_2_0 following the protocol described in phase M2 of the cell viability / cytotoxicity test using MTT.

*PHASE E3 - Treatment of cells with NPs, STS and Rap*

- After 24 h from the seeding, CTC medium was substituted with SFC medium, and A549 cells were treated with ZnO-NPs (1, 5 and 20 μg/cm^2^), and continuously monitored for at least 144 hours.

- The necessary dilutions of NPs in SFS + 1% of H_2_0 were prepared by taking 100 μl from thecolumns 5, 6, and 7 of the dilution plate (Figure 1, Table 1), following the protocol described in step M2 of the testcell viability/cytotoxicity by MTT.Since the area of ​​the wells of the array is approximately 0.5 cm^2^, a dilution factor is appliedof NP equal to 0.5.Thus for the treatment of the ECIS chambers were inoculated in each well as follows:

Total 10 μgNPs to obtain the concentration of 20 μg/cm^2^

Total 2.5 μg NPs to obtain the concentration of 5 μg/cm^2^

Total 0.5 μg NPs to obtain the concentration of 1 μg/cm^2^

- For apoptosis or autophagy positive controls, wells were treated with Staurosporine (1 μM) or Rapamycin (10 μM), respectively.

**CELLULAR VITALITY/CYTOXICITY TEST USING NEUTRAL RED (PHASES R1- R7)**

Viability tests using neutral red were performed to compare the resultsobtained with the MTT test and the ECIS test.

*PHASE R1 - Preparation of cell culture in flat-bottom 96-well treatment plates*

In phase R1 the experimental procedures described for phase M1were followed.

*PHASE R2 - Preparation of NP dilutions in the flat-bottom dilution plate*

In phase R2, the experimental procedures described for phase M2 were followed.

*PHASE R3 - Treatment of cells with NP, Staurosporin and Rapamycin*

In phase R3, the experimental procedures described for phase M3 were followed.

*PHASE R4 - Preparation of the solutions for the Neutral Red test*

The protocol according to Repetto G et al., 2008was followed.

Accordingly:

- 12 ml of Neutral Red reagent were prepared, starting from a stock solution (4 mg/ml)mixing 0.12 ml with 12 ml of medium (SFS), to obtain a solution equal to 40μg/ml. Incubation overnight at the cell growth temperature.

Note: This solution must be prepared the day before use.

*PHASE R5 - Check measuring equipment*

To verify the performance of the microplate reader, a bottom-flat 96-well plate was used, with each well filled with equal volumes of Neutral Red Assay Reagent.

- 10 ml of absorption solution was poured into a V-shaped tank (the plastic containerwhere to put the liquids to be taken with the multichannel must be sterilized before use).

-Using a 6-channel multichannel pipette, 200 μl was added to each well of theplate to be read. The absorbance measurements of each well should not showoutliers. In case of abnormal values, a new plate should be prepared and the procedurerepeated.

*PHASE R6 - Execution of the Red Neutral test*

- At the end of treatments, the individual wells of the plate were checked with a phase contrastmicroscope for morphology, percentage of confluence, and possible contamination(in which case the plates were discarded).

- Treatments were carefully removed with a multichannel pipette.

- With a multichannel pipette, 100 μl of neutral red reagent (40μg/ml) were added in each well of the treatment plate containing A549 cells, by avoidingthe formation of air bubbles.

- The wells were checked for the presence of air bubbles, and removed using a needlesyringe.

Note: air bubbles can significantly affect absorbance measurements made with a plate reader.

- The plate was incubated at 37 °C for 2 h in a humidified atmosphere at 5% CO_2_.

- The presence of neutral red precipitates in the cells was checked under a contrast phase microscope.

- The neutral red reagent was removed.

- A wash was carried out with 150 μl of PBS.

- 150 μl of decoloring solution (consisting of 50% ethanol at 96%, 49%deionized water, 1% glacial acetic acid) was addedboth in the wells delimited by the blue box (control and treated cells) both in the wells withoutcells (column 2, blank) of the flat-bottom 96-well treatment plate (Figure 2).

Note: It is recommended to lysate the cells and dissolve neutral red crystals by carefully pipetting the sample in each well.

- The plate was then incubated at 37 °C for 10 minutes, in a humidified atmosphere at 5% CO2.

- The absorbance at 540 nm was finally measured with a microplate reader.

Note: the amount of colored product is proportional to the number of live cells. If a treatment causes cell death, decreases absorbance.

*PHASE R7 - Data analysis*

The procedure was similar to that described in phase M7 of the MTT test.

**ASSESSMENT OF THE PROLIFERATIVE CAPACITY OF A549 BYCLONING EFFICIENCY TEST AFTER TREATMENT WITH NPs, STAUROSPORINE AND RAPAMICIN (PHASES K1-K7)**

In this experimental phase, the clonogenicity test was carried out to evaluate the effect of NPson the proliferative capacity of A549 cells, by comparing it with STS and Rap, inducers of apoptotic and autophagic death, respectively.

*PHASE K1 - Preparation of cell culture in 24-well treatment plates*

K1.1 Preparation of complete tissue culture medium (CTC)

The procedure followed in this phase is the same as that described in phase M 1.1.

K1.2 Preparation of the single cell suspension

The procedure followed in this phase is the same as that described in phase M1.2.

K1.3 Count of cells contained in the suspension

The procedure followed in this phase is the same as that described in phase M1.3.

K1.4 Cell seeding

For comparison with the previous experiments, 150,000 cells were seeded in each well(surface area of ​​the 24 well plate is equal to 1.9 cm^2^), to maintain NP/surface and NP/cells ratio used in the other texts.

N.B.: each point has been made in duplicate

- After settling at room temperature under the sterile flow for 15 min, the plate iswas gently transferred to a humidified incubator, at 37 °C with 5% CO2.

*PHASE K2 - Preparation of NP dilutions in the flat-bottom dilution-plate*

This procedure was performed in a Biohazard laminar flow hood to minimize the medium contamination with bacteria or fungi.The standard NP solutions used in the tests come from Sigma Aldrich. They are solutions

containing 1.7 g/ml of NP in H_2_O, stored at 25 °C.It was decided to conduct also this experiment in serum-free medium (SFS) rather than in CTC, as fromcurrent literature data the serum tends to create a coating on the NPs (“protein corona” effect).

- The medium was prepared without the addition of 10% FBS (SFS), but containing 1% of sterile H_2_O,and mixing by inversion.

- SFS medium (with added antibiotics) containing 1% H_2_O was used as a vehicle to dissolve the NPs.

- 1 ml of NP stock solution was pipetted under sterile conditions, and put in a 15 ml polypropylene tube by a 2 ml pipette.

- The tube containing the suspension of NP was sonicated for 30 min, immersed in H_2_O eice.

- NP dilutions were prepared immediately prior to cell treatment. Thescheme containing the repeated columns of serial dilutions of the NPs, is shown in Table 2.

- The 1g/ml solution was prepared separately by making a 1:1.7 dilutionin SFS.

- At the end the solution was transferred to column 1 of the flat-bottom96-well assay plate (Figure 3) following the scheme in Table 2.


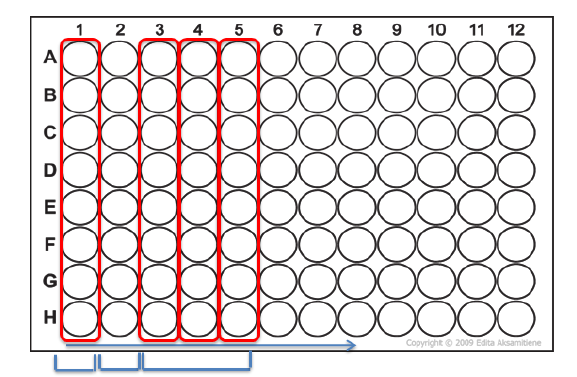


**Figure 3 – Flat-bottom 96-well treatment plate**

*PHASE K3 - Treatment of cells with NP, Staurosporin and Rapamycin*

K 3.1 Treatment of cells with NPs

The plates with seeded cells were treated in the following ways:

- after incubation for 24 h, cells were observed under a phase contrast microscope to check cell adhesion, density and possible contamination;

- The medium (1 ml) was then removed and 1ml SFS was added in each well.

- The cells were then treated by adding 3.8 microliters from columns 3, 4, and 5 (Figure 3) in each well, (i.e. 3.8 μl in 1 ml final volume). Inparticular, they were collected respectively: 38 μg (column 3), 9.5 μg(column 4), and 1.9 μg of NPs (column 5), to be administered on the surface of 1.9 cm^2^ of eachwell of 24 well-plate to obtain a concentration corresponding to 20 μg/cm^2^, 5 μg/cm^2^, and 1 μg/cm^2^, respectively.

|  | Column1 | Column2 | Column3 | Column4 | Column5 |
| --- | --- | --- | --- | --- | --- |
|  | 1g/ml | (1:10) | (1:10) | (1:4) | (1:5) |
| A |  | 20 μl Col 1  +180 μl SFS | 20 μl Col 2  +180 μl SFS) | 50 μl Col 3  +150 μl SFS | 40 μl Col 4  +160 μl SFS |
| B |  | 20 μl Col 1  +180 μl SFS | 20 μl Col 2  +180 μl SFS | 50 μl Col 3  +150 μl SFS | 40 μl Col 4  +160 μl SFS |
| C |  | 20 μl Col 1  +180 μl SFS | 20 μl Col 2  +180 μl SFS | 50 μl Col 3  +150 μl SFS | 40 μl Col 4  +160 μl SFS |
| D |  | 20 μl Col 1  +180 μl SFS | 20 μl Col 2  +180 μl SFS | 50 μl Col 3  +150 μl SFS | 40 μl Col 4  +160 μl SFS |
| E |  | 20 μl Col 1  +180 μl SFS | 20 μl Col 2  +180 μl SFS | 50 μl Col 3  +150 μl SFS | 40 μl Col 4  +160 μl SFS |
| F |  | 20 μl Col 1  +180 μl SFS | 20 μl Col 2  +180 μl SFS | 50 μl Col 3  +150 μl SFS | 40 μl Col 4  +160 μl SFS |
| G |  | 20 μl Col 1  +180 μl SFS | 20 μl Col 2  +180 μl SFS | 50 μl Col 3  +150 μl SFS | 40 μl Col 4  +160 μl SFS |
| H |  | 20 μl Col 1  +180 μl SFS | 20 μl Col 2  +180 μl SFS | 50 μl Col 3  +150 μl SFS | 40 μl Col 4  +160 μl SFS |
|  | 1000μg/μl | 100 μg/μl | 10 μg/μl | 2.5 μg/μl | 0.5 μg/μl |

**Table II – NP dilution scheme for cloning efficiency**

K3.2 Treatment of cells with Staurosporine (STS)

The cells were also treated with Staurosporine (1 µM), as positive control for apoptosis.An aliquot was taken from a 1mM mother and administered directly into the cells to havea concentration equal to 1μM (1 μl in 1 ml, dilution 1: 1000).

K3.3 Treatment of cells with Rapamycin (Rap)

The cells were also treated with Rapamycin (10 µM), as positive control for autophagy.

An aliquot was taken from a 1 mg/ml (1 mM) mother and administered directly to thecells to have a concentration equal to 10 μM (10 μl in 1 ml, dilution 1:100).

- The plates treated for24, 48, 72, and 144 h were treated according to the scheme in Figure 4


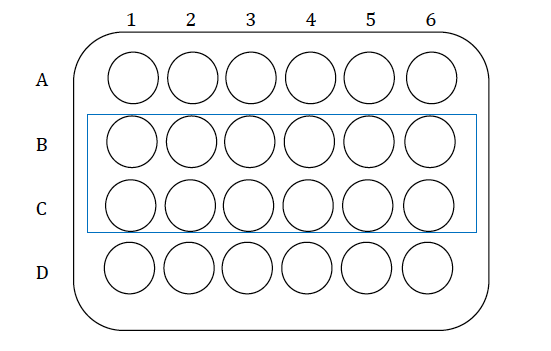


**Figure 4 – Flat-bottom 24-well treatment plate**

Blue square

Column 1: CTR

Column 2: ZnO 20 mg/cm^2^

Column 3: ZnO 5 mg/cm^2^

Column 4: ZnO 1 mg/cm^2^

Column 5: Rapamycin 10 µM

Column 6: STS 1 µM

-After the treatment, the cells were observed by a phase contrast microscope to check the state of adhesion and, then, transferred to the humidified incubator, at 37 °C with CO_2_ al 5%.

*PHASE K4 - Plating cells for clonogenic test*

At the end of exposure to NPs, Staurosporin and Rapamycin, each well of theplates were checked under a microscope for morphology, state of health, percentage ofconfluence and possible contamination (in which case the plates were discarded). We proceededthen to the cell plating for the clonogenic assay.

K4.1 Preparation of complete tissue culture medium

The medium used was RPMI-1640, added with L-glutamine, penicillin (50 U/ml),streptomycin (50 μg/ ml), and 10% FCS. The completed tissue culture medium (CTC) wasstored at 4 °C.

K4.2 Preparation of the single cell suspension

Cell suspensions from the different treatments were prepared asfollows:

- CTC, EDTA and trypsin were pre-heated at 37 °C in a thermostatedwater bath.

- After removing the treatment medium, a solution of EDTA (10mM, 0.5 ml) was added, shaking gently for a few seconds.

- A trypsin solution (0.25%, 0.5 ml) was also added, stirring slightly for a fewseconds, then the culture was transferred to the humidified incubator at 37 °C with 5% CO_2_,and incubated for 3-5 min.

- After checking cellsbylight microscopy (if they became rounded in shape and detached from thebottom of the flask), 2ml of CTC (final volume 3ml) was added by gently pipetting thecells to disperse them homogeneously.

Note: it is particularly important to obtain a single cell suspension to make an appropriate cell count.

K4.3 Count of cells contained in the suspension

The count was carried out as follows:

- 20 μl of cell suspension, obtained in the K2 phase, was transferred with a micropipette ina sterile eppendorf tube and mixed with 20 μL of a Trypan blue (TB) solution (dilution 1: 2).

- 10 μL of the obtained suspension were then loaded into aNeubauer chamber.

- The cells in the four quadrants of the chamber were then counted under a phase contrast microscope. The

count was repeated twice.

The calculation of the number of cells was carried out by applying the following formula:

N = {[(a + b) / 8] * 10,000 * DF}

Where it is:

N = number of cells/ml

a = number of cells counted in the 4 quadrants of the first chamber

b = number of cells counted in the 4 quadrants of the second chamber

8 = number of quadrants counted

*10,000 (10^4^) = conversion factor for the chamber volume

*DF = dilution factor of the cell suspension (generally equal to 2);

Note. Both negative (viable) and positive (dead) TB cells were counted.

K4.4 Cell seeding

Only viable cells(TBnegative) were considered for the seeding of the clonogenic test. For each sample, three Petri dishes with a diameter of 6 cm were prepared. For theseeding the following procedure was followed:

- for each sample a cell suspension containing 3x10^3^ cells was first prepared in15 ml of CTC.

- Then, 5 ml of cell suspension were seeded (i.e. 10^3^ cells)in each 6 cm-diameter Petri dish.

Note: to optimize the homogeneity in the preparation, it is important to mix the cell suspension. This minimizes sedimentation of cells at the bottom of the tank.

- The plates were then transferred to the humidified CO_2_incubator at 37 °C for thecolony growth (10-14 days).

*PHASE K5 - Preparation of methylene blue solutions*

The methylene blue solution used to stain the colonies was prepared withas follows:

- 0.5g of methylene blue powder was weighed in a precision balance.

- They were then dissolved in 400 ml of 80% ethanol to obtain a saturated solution which waskept at room temperature for at least 24 h.

- Before use, the solution was filtered with the bibulous paper.

*PHASE K6 - Coloring of the colonies*

At the end of the incubation period, the colonies were stained as follows:

- the medium was gently removed from the 6 cm Petri dishes.

- After a PBS wash, the colonies were fixed with 95% ethanol for 10 min.

- Once the fixative was discarded, the colonies were stained with methylene blue in 80% ethanol (for at least 2 h).

Note. Check the staining of colonies before removing the dye

- They were finally washed with distilled water and left to dry.

.

*PHASE K7 - Data analysis*

For the determination of cell survival the following procedure was performed:

- the colonies were counted with the ImageJ Program.

- The mean values ​​of each single treatment were then calculated for each experimental setcarried out in triplicate.

- The values ​​of the treated wells were then compared with the values ​​of the untreated wells.

The resulting values ​​represent the fraction of cells that survive after treatment and canbe expressed as cell survival percentage with the following formula:

% cell survival = [colonyN° of treated cells / colony N° of untreated cells x 100]

For each sample and for each experimental set the percentage ofcell deathwas calculated as:

% cell death = number of TB positive cells / number of TB positive cells + number of TB negative cells) * 100

**Critical issuesand suggestion**

- The appropriate type of ECIS array should be identified to obtain useful output of cytotoxicity data.

- For an optimal and comparable data output with the MTT test, it is necessary to carefully evaluate the number of cells seeded in the arrays for the ECIS apparatus (in our case from 4,000 to 40,000).

- It is also considered appropriate to seed and treat the cells in the arrays already installed in the ECIS reader to monitor any variations induced by the seeding and treatment operations.

- It seems appropriate to determine the optimal treatment conditions in terms of concentrations ofNP and incubation times.

- It seems appropriate to verify the influence of the presence of the serum in the treatment medium onachieved results.

- When comparing with other tests it is advisable to relate both the number of cells and the concentration of particles with the surface area used (cells/cm^2^; NPs μg/cm^2^).

- After finalized the concentrations and treatment times of NPs, evaluate the opportunityto insert a standard reference substance as an inducer of cell death.

- It is advisable to confirm the type of cell death with biochemical tests and ultrastructural cell pathology studies.
